# Serendipitous Discovery of Short Peptides Dimethylammonium Salt Promoting Neural Differentiation

**DOI:** 10.1002/psc.70124

**Published:** 2026-08-20

**Authors:** Sadiq Noor Khan, Maha Shahid, Shahzad Nazir, Anila Bashir, Sumbla Sheikh, Shabana Usman Simjee, Marc Maresca, Farzana Shaheen

**Affiliations:** ^1^ Third World Center for Science and Technology, H. E. J. Research Institute of Chemistry, International Center for Chemical and Biological Sciences University of Karachi Karachi Pakistan; ^2^ H. E. J. Research Institute of Chemistry, International Center for Chemical and Biological Sciences University of Karachi Karachi Pakistan; ^3^ Institute for Medical Virology and Epidemiology of Viral Diseases University of Tübingen Tübingen Germany; ^4^ Dr. Panjwani Center for Molecular Medicine and Drug Research, International Center for Chemical and Biological Sciences University of Karachi Karachi Pakistan; ^5^ Aix‐Marseille Univ CNRS, Centrale Marseille, iSm2 Marseille France

## Abstract

The synthesis and in vitro neuronal growth support activity of a novel series of heptapeptides (**1**–**19**) are reported here. Structural studies of the peptide **3** (LSAVTFG.DMA) revealed an unexpected dimethylammonium (DMA) salt adduct formation localized at the *C*‐terminal glycine residue. DOSY NMR analysis revealed the presence of two distinct species exhibiting different diffusion coefficients, allowing the differentiation between the peptide and the DMA adduct. Peptide **3** promoted neural differentiation at 100 μM concentration. To identify residues crucial for biological activity, we conducted L‐ and D‐alanine scans with salt and nonsalt form of peptide **3,** which resulted in the synthesis of 16 peptide analogs. Findings from the alanine scan revealed that the lead peptides L1A.DMA (**13**) and S2A.DMA (**15**) exhibited significant neural progenitor cells or neural stem cells (NPCs/NSCs) differentiation activity. These peptides promote neuronal survival, morphology recovery, increase cell proliferation, and preserve neuronal network integrity under OGD/R and hypoxia/R insult in an in vitro model of ischemic stroke. These results represent the first synthetic approach to new neuromodulatory peptides and deliver important structure–activity relationship (SAR) insights to guide the development of bioactive peptides.

## Graphical abstract

Design and synthesis of new heptapeptides exhibiting significant neuroprotection and structure–activity relationship (SAR) insights to guide the development of bioactive peptides.

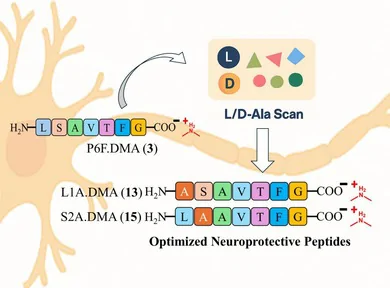

## 1 Introduction

Neurodegenerative disorders and hypoxic–ischemic brain injury are two major global health problems. They are characterized by progressive neuronal loss and a subsequent loss in brain function as well as cognitive decline [1, 2]. Diseases such as Alzheimer, Parkinson, cerebral ischemia and stroke are strongly associated with oxidative stress, excitotoxicity, mitochondrial dysfunction, and neuronal apoptosis [3, 4, 5]. Despite significant advances in neuroscience, effective therapeutic strategies that can simultaneously protect neurons and promote neuronal regeneration remain limited.

Compared to small molecule drugs, peptide‐based drugs generally show higher target selectivity and specificity, which can lead to more precise effect on the onset and progression of diseases, resulting in enhanced therapeutic efficacy and reducing a higher risk of side effects [6]. Bioactive peptides have a lot to offer in the treatment of inflammation, oxidative stress, neuronal regeneration, localized ischemia, and the blood–brain barrier. These peptides could be used alone or in combination with traditional pharmacological treatments to improve the management of diseases associated with Alzheimer's and brain disorders [7, 8]. In addition, these peptides can influence neuronal survival signaling, reduce reactive oxygen species, and enhance cellular resilience under stress conditions [9, 10]. Various bioactive neuroprotective peptides studied in OGD/R and hypoxia/reoxygenation injury models are reported in literature [11, 12, 13, 14, 15, 16].

For evaluating potential neuroprotective agents using ischemia‐like conditions, in vitro models such as oxygen–glucose deprivation and reoxygenation (OGD/R) and hypoxia/reoxygenation systems have been widely used. The pathological environment of stroke by inducing energy failure, oxidative stress, and neuronal injury closely mimics these in vitro models [17, 18, 19, 20]. In this study, primary neuronal cultures acquired from neonatal rodent brain tissue, especially cortical neurons from pups of Wistar rat, serve as a potentially reliable and effective experimental model to study neuronal injury and regeneration.

In addition to neuroprotection, the ability of compounds to promote neurogenic activity and neuronal differentiation is of increasing interest, as restoration of neuronal networks is essential for functional recovery after brain injury [21, 22, 23]. Therefore, screening of novel test compounds for both neuroprotective and neurogenic potential is crucial for identifying dual‐acting therapeutic candidates.

In continuation of our ongoing efforts to synthesize and biologically evaluate the cyclic peptide natural products and their corresponding linear precursors, we serendipitously identified some linear heptapeptides with potential neuroprotective properties. These peptides were further subjected to in vitro screening to assess their neurogenic and neuroprotective activities against oxygen–glucose deprivation/reoxygenation (OGD/R) and hypoxia‐induced injury models using primary cortical neurons. The neuronal viability and functional integrity were assessed using standard cell‐based assays. This work aims to identify peptide‐based candidates that can support neuronal survival and promote neurogenic responses under pathological conditions.

## 2 Materials and Methods

### 2.1 Preparation of Peptides

The linear precursor of *annomuricatin* F [24] and its analogues (**1–19**) were manually synthesized on Wang resin (1.4 mmol/g loading) using the standard *N*α‐Fmoc peptide synthesis protocol, as previously described in our earlier work [25]. The ester bond formation in the first coupling was facilitated by the addition of DMAP. Coupling reactions were performed using 3 equivalents of protected amino acids (Fmoc‐AA), OxymaPure, and DIC in a 1:1:1 ratio dissolved in DMF. The desired peptide chains were extended through successive cycles of amino acid coupling reactions. Deprotection of Fmoc group was performed using 20% of 4‐methylpiperidine in DMF (1 × 20 min).

#### 2.1.1 Obtaining Salt‐Free Peptides

**SCHEME 1:**
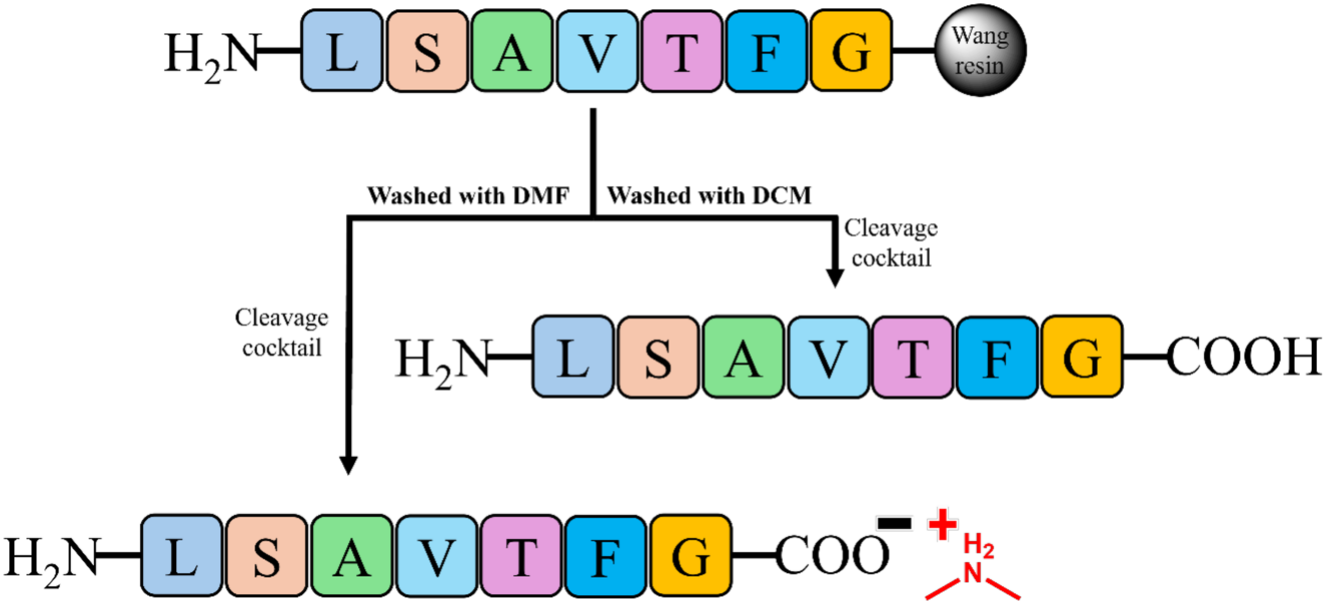
Peptides salt formation through washing with DMF.

The peptidyl resin was washed with dichloromethane (DCM). Cleavage and global deprotection of peptides were achieved by treating the peptide‐bound resin with a TFA cocktail (95% TFA and 5% H_2_O) for 2 h at ambient temperature. TFA was removed under vacuum via rotary evaporation, followed by drying of the crude peptides via lyophilization (Scheme 1).

#### 2.1.2 Obtaining Salt‐Associated Peptides

The peptidyl resin was washed with *N,N*‐dimethylforamide (DMF). Cleavage and global deprotection of peptides were achieved by treating the peptide‐bound resin with a TFA cocktail (95% TFA and 5% H_2_O) for 2 h at ambient temperature. TFA was removed under vacuum via rotary evaporation, followed by drying of the crude peptides via lyophilization (Scheme 1).

#### 2.1.3 Purification of Crude Peptides

The peptides were then purified using a puriFlash 5.125 reversed‐phase preparative high‐performance liquid chromatography (RP‐HPLC) system controlled by Interchim software. Purification was performed using a C18 column (PFB15C18XS‐250/212) with 15‐μm particle size and 100‐Å pore size. UV detection at 214 nm was employed, and the crude peptides were eluted isocratically using 60% acetonitrile in deionized water at ambient temperature. The mobile phase was delivered at a flow rate of 4.0 mL/min, with the eluent containing 0.1% (v/v) TFA. Fractions were analyzed using ultra‐performance liquid chromatography (UPLC) (Agilent 1260 Infinity Diode Array) equipped with a C‐4 reverse‐phase analytical column (5 μm, 2.8 mm), employing a mobile phase of acetonitrile/water (60:40) containing 0.08% TFA.

#### 2.1.4 Characterization

Low‐resolution and high‐resolution fast atom bombardment (LR‐FAB and HR‐FAB) mass spectra were acquired using QSTAR XL MS/MS systems (Agilent, USA). NMR spectra were recorded using Bruker Avance spectrometers operating at 600 and 800 MHz. The NMR data were collected at 25°C.

### 2.2 Animals

The rat pups of Wistar strain were procured from the Animal House Facility at the International Center for Chemical and Biological Sciences (ICCBS), University of Karachi. All experimental procedures followed were in accordance with the ethical guidelines provided by the Scientific Advisory Committee on Animal Care, Use, and Standards, ICCBS (*Protocol #* 2021‐014).

### 2.3 Isolation and Culturing of Primary Cortical Cells

The primary cortical cells were isolated from the 1‐ to 3‐day‐old neonatal rat pup. The isolation of the cortex from brain samples was carried out under laminar flow hood Class II, Type A2 Biohazard Safety Cabinet. Briefly, after decapitation, the cranium was exposed followed by the isolation of brain tissue, which was transferred to a petri dish and washed with sterile 1× phosphate buffer saline (PBS). Following harvestation, the meninges were removed from cerebral cortex and a portion of cortex was taken from each lobe. The separated tissues were homogenized and transferred into a sterile Falcon tube containing 1 mL of trypsin–EDTA 0.05% solution and incubated for 3 min in 5% CO2 humidified chamber at 37°C for tissue digestion. After trypsinization, fresh medium was added and fine suspension was prepared by gently pipetting the tissue mixture. Once the cell suspension was formed, it was centrifuged at 241 g for 5 min. Following centrifugation, supernatant was removed and pellet was resuspended in 1 mL fresh complete DMEM culture media (containing FBS 10%, sodium pyruvate 1 mM, penicillin–streptomycin 100 U/mL, and amphotericin B 0.25 μg/mL). After checking the cell count and viability, cell suspension was then transferred to a tissue culture treated flask (75 cm2) and incubated with 5% CO2 at 37°C. At this stage, the cells will be considered as P0. Cells in the flasks were observed daily for gross morphology until they were confluent and ready to use for MTT assay.

### 2.4 Screening of Peptides for In Vitro Neuronal Growth Support Activity

The 100 mM stock solution of each peptide was prepared in DMSO, and working concentrations were further prepared by diluting in the culture medium. The effect of each compound on cell viability was analyzed by employing the MTT assay. To perform the MTT assay, the cultured cells were trypsinized with 0.05% trypsin–EDTA solution followed by centrifugation at 241 g for 5 min. After centrifugation, the supernatant was removed, and the pellet was resuspended in fresh 1 mL culture media. The cells were seeded in a density of 9000 cells/200 μL/well in a flat bottom 96‐well plate and were incubated at 37°C in humidified 5% CO_2_ incubator for 48 h to allow cell attachment and stabilization of neuronal morphology, defined by cell body integrity, neurite extension, and overall cellular architecture in addition to the observation of achieving at least 70% confluency. Following incubation time, the medium was aspirated and cells were treated with 50 and 100 μM concentrations of test compounds for 48 h. The cells with only medium served as control and for a vehicle control, cells were treated with 0.1% DMSO in complete media. The same complete culture media without cells served as a blank. After treatment duration, medium was removed and 0.5 mg/mL (100 μL) of MTT dye was added in each well in the dark followed by incubation at 37°C in 5% CO_2_ for 4 h. Following incubation, dye was replaced with stopping solution containing 100 μL of DMSO and absorbance was measured at 570‐nm wavelength using spectrophotometer. The percent proliferation rate was calculated by using the formula mentioned below:

$$\text{Percentage Proliferation Rate}=\left[\left(\mathrm{At}-\mathrm{Ab}\right)/\left(\mathrm{Ac}-\mathrm{Ab}\right)\right]\times 100$$

Absorbance value of test compounds = At.

Absorbance value of blank = Ab.

Absorbance value of control = Ac.

### 2.5 In Vitro Oxygen Glucose Deprivation and Hypoxia Insult

Oxygen glucose deprivation (OGD) and hypoxia experiments were carried out as previously described with slight modifications [26]. After pilot screening of the compounds through MTT assay, the OGD/R experiment was commenced. Cells were trypsinized, centrifuged, counted, and seeded in 96‐well plate as mentioned before in Section 2.3. The cells were treated with the doses of selected compounds for 48 h based on the results of MTT assay. Following treatment, the cells were subjected to OGD and hypoxic insult. Briefly, the culture medium is replaced with the glucose free DMEM and cells were transferred to hypoxia chamber (New Brunswick Galaxy 48 R) supplemented with 94% N_2_/5% CO_2_/1%O_2_ at 37°C for 3 h OGD insult. In the same experiment, a set of experimental groups were subjected to hypoxia insult by replacing the media with fresh complete DMEM and transferred to hypoxia chamber supplemented with same gas mixture as mentioned before for 3 h. Only cells with and without glucose free media with similar incubation conditions served as OGD and hypoxia disease control groups. Additionally, only cells with glucose (control group) media with normal incubation conditions, that is, 95% air and 5% CO_2_ incubator at 37°C were treated for the same time frame set for the OGD and hypoxia insult. At the end of OGD and hypoxia insult, 100 μL of complete media was added in each well and reintroduced to the normal incubator conditions for 21 h. Following reoxygenation phase, the cells were analyzed for morphological alterations and percent rate of proliferation was determined by MTT assay.

### 2.6 Statistical Analysis

The data in this study was statistically analyzed by using one‐way ANOVA, further post hoc analysis used were Dunnett's test and Bonferroni's post hoc test using Statistical Package for the Social Sciences (SPSS) software v20. Dunnett's test was employed where the mean of different test groups was compared only with control group. When all possible means are compared then Bonferroni's post hoc was applied. The experimental data of the study was represented as mean ± standard error of mean (S.E.M). Each experiment was performed thrice and the significance of data was expressed as *p ≤ 0.05, **p ≤ 0.01, and ***p ≤ 0.001.

## 3 Results

### 3.1 Characterization of the Peptides

The characterization of the peptides is summarized in Table 1. Following purification by preparative HPLC, all peptides exhibited purities exceeding 95%, and their chemical structures were confirmed through mass spectrometry, ^1^H‐NMR, ^13^C‐NMR, and 2D‐NMR (see Supporting Information Figures for details).

**TABLE 1:** Structural characteristics of the compounds.

S. no.	Abbreviation	Structures	Molecular formula	Mass (Da)	
				Calculated	Observed
**1**	LP	LSAVTPG	C_28_H_49_N_7_O_10_	[M + H]^+^: 644.3619	[M + H]^+^: 644.3625
**2**	P6F	LSAVTFG	C_32_H_51_N_7_O_10_	[M + H]^+^: 694.3776	[M + H]^+^: 694.3811
**3**	P6F.DMA	LSAVTFG.DMA	C_32_H_51_N_7_O_10_^−^.C_2_H_8_N^+^	[M + H]^+^: 694.3776	[M + H]^+^: 694.3789
**4**	L1 **A**	**A** SAVTFG	C_29_H_45_N_7_O_10_	[M + H]^+^: 652.3306	[M + H]^+^: 652.3328
**5**	L1 **A** .DMA	**A** SAVTFG.DMA	C_29_H_45_N_7_O_10_^−^.C_2_H_8_N^+^	[M + H]^+^: 652.3306	[M + H]^+^: 652.3335
**6**	S2 **A**	L **A** AVTFG	C_32_H_51_N_7_O_9_	[M + H]^+^: 678.3827	[M + H]^+^: 678.3856
**7**	S2 **A** .DMA	L **A** AVTFG.DMA	C_32_H_51_N_7_O_9_^−^.C_2_H_8_N^+^	[M + H]^+^: 678.3827	[M + H]^+^: 678.3866
**8**	V4 **A**	LSA **A** TFG	C_30_H_47_N_7_O_10_	[M + H]^+^: 666.3463	[M + H]^+^: 666.3457
**9**	T5 **A**	LSAV **A** FG	C_31_H_49_N_7_O_9_	[M + H]^+^: 664.3670	[M + H]^+^: 664.3686
**10**	F6 **A**	LSAVT **A** G	C_26_H_47_N_7_O_10_	[M + H]^+^: 618.3463	[M + H]^+^: 618.3479
**11**	G7 **A**	LSAVTF **A**	C_33_H_53_N_7_O_10_	[M + H]^+^: 708.3932	[M + H]^+^: 708.3916
**12**	L1A	ASAVTFG	C_29_H_45_N_7_O_10_	[M + H]^+^: 652.3306	[M + H]^+^: 652.3348
**13**	L1A.DMA	ASAVTFG.DMA	C_29_H_45_N_7_O_10_^−^.C_2_H_8_N^+^	[M + H]^+^: 652.3306	[M + H]^+^: 652.3292
**14**	S2A	LAAVTFG	C_32_H_51_N_7_O_9_	[M + H]^+^: 678.3827	[M + H]^+^: 678.3781
**15**	S2A.DMA	LAAVTFG.DMA	C_32_H_51_N_7_O_9_^−^.C_2_H_8_N^+^	[M + H]^+^: 678.3827	[M + H]^+^: 678.3799
**16**	V4A.DMA	LSAATFG.DMA	C_31_H_49_N_7_O_9_^−^. C_2_H_8_N^+^	[M + H]^+^: 666.3463	[M + H]^+^: 666.3433
**17**	T5A.DMA	LSAVAFG.DMA	C_31_H_49_N_7_O_9_^−^. C_2_H_8_N^+^	[M + H]^+^: 664.3670	[M + H]^+^: 664.3647
**18**	F6A.DMA	LSAVTAG.DMA	C_26_H_47_N_7_O_10_^−^. C_2_H_8_N^+^	[M + H]^+^: 618.3463	[M + H]^+^: 618.3445
**19**	G7A.DMA	LSAVTFA.DMA	C_33_H_53_N_7_O_10_^−^. C_2_H_8_N^+^	[M + H]^+^: 708.3932	[M + H]^+^: 708.3976

*Note:* Underlined and bold letters indicate D‐amino acid.

### 3.2 Identification of Peptides as DMA Adduct

**SCHEME 2:**
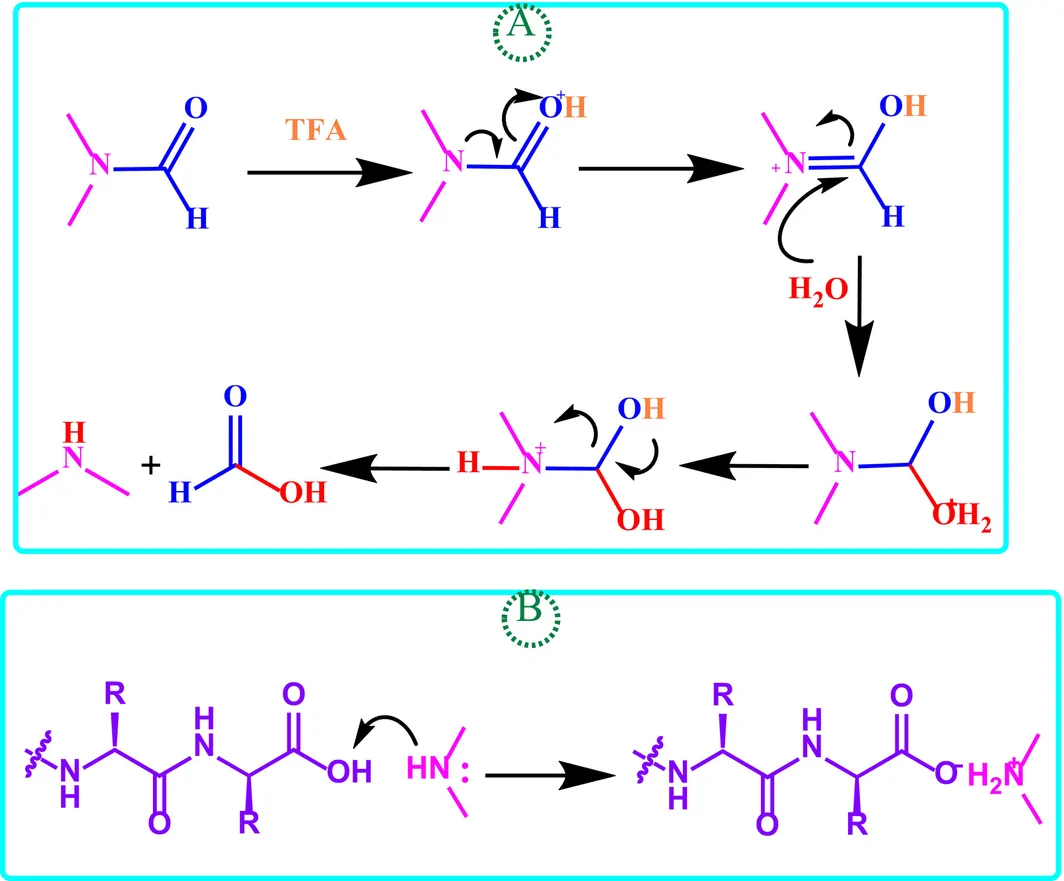
Proposed mechanism of dimethyl amine (DMA) salt formation.

DMF undergoes acid‐catalyzed hydrolysis in the presence of trifluoroacetic acid (TFA) and trace water, producing dimethylamine and formic acid. The released dimethylamine is quickly protonated by *C*‐terminal peptide carboxylic acid group, producing the dimethylammonium cation. The resulting dimethylammonium and carboxylate ions spontaneously associate to form a stable salt (Scheme 2). The in situ formation of dimethylammonium carboxylate underscores that DMF functions as more than merely a solvent [27, 28], contributing actively to the reaction outcome.

To determine the structure of the peptide **3** (P6F.DMA), we performed detailed 1D and 2D NMR analyses on both the peptide and its corresponding dimethylammonium (DMA) salt (see Figures S11–19). FAB (+)‐MS/MS analysis of peptide **3** (P6F.DMA) verified the anticipated [M + H]^+^ ion of P6F (LSAVTFG), while the molecular weight of DMA could not be established from FAB‐MS data.

**FIGURE 1:**
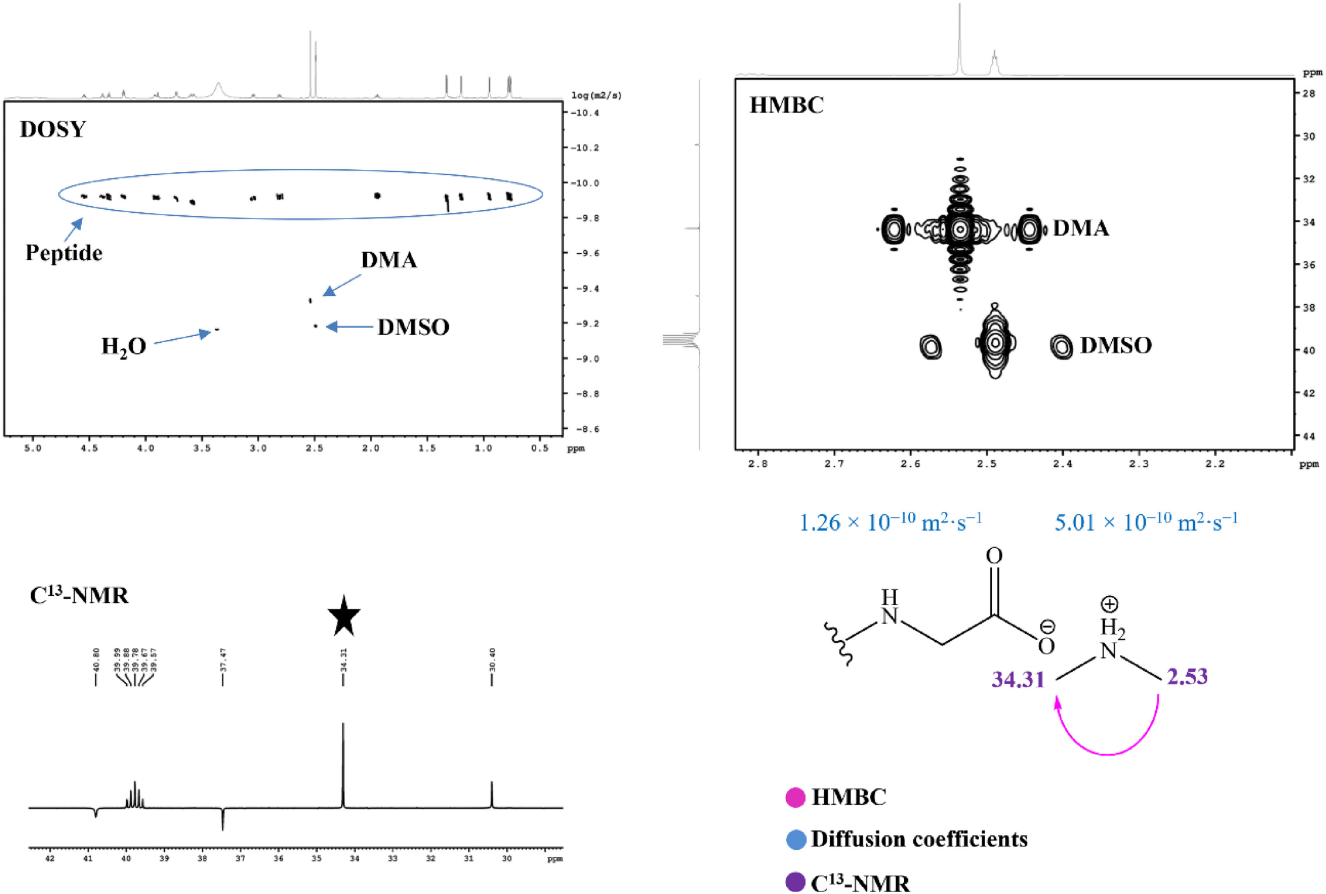
Structural characterization of peptide salt by NMR spectroscopy. ^13^C NMR spectrum with the diagnostic DMA group chemical shift highlighted (★) and HMBC correlations confirming the DMA structure and DOSY spectra confirming the mixtures of two.

^1^H NMR analysis of peptide **3** (P6F.DMA), in DMSO‐*d6* showed an extra singlet at δ 2.53 ppm, near the solvent peak (Figure 1), which was not present in the spectrum of the free peptide **2**. HSQC analysis showed that this signal was directly correlated with a carbon resonance at δ 34.3 ppm. DEPT‐135° and DEPT‐90° experiments verified the presence of methyl carbon, which appeared in DEPT‐135° but was absent in DEPT‐90°. HMBC spectrum revealed that the singlet at δ 2.53 ppm displayed three‐bond (^3^J‐CH) correlations with another methyl carbon at the same chemical shift, consistent with the presence of a +NH_2_(CH_3_)_2_ group.

We propose that DMA interacts with the *C*‐terminal Gly residue of P6F (LSAVTFG) to form a salt, most likely via protonation of the carboxylic acid group (pKa 3–4). DOSY NMR analysis of P6F·DMA (**3**) showed diffusion coefficients (D) 1.26 × 10^−10^ m^2^·s^−1^ and 5.01 × 10^−10^ m^2^·s^−1^ of two distinct species, corresponding to the peptide and DMA, respectively. The marked difference in diffusion coefficients enabled clear distinction between the peptide and the dimethylammonium counterion in solution.

### 3.3 In Vitro Screening of Peptides to Determine Neural Differentiation Activity

The percent rate of proliferation of all compounds was carried out through MTT assay on primary cortical cultures. It is to be noted here that the experiments were not performed on terminally differentiated neurons, but rather on a neural stem/progenitor cell population (NSCs/NPCs), which retains the capacity to proliferate and differentiate were studied. Following treatment with the 50 and 100 μM concentrations of test peptides for 48 h, the cells of each test group were observed for the morphological characteristics based on their cell viability, proliferation and differentiation activity. The absorbance was measured and percent proliferation was determined. The percentage of proliferation along with S.E.M. and significance values of each peptide is displayed in Table 2.

**TABLE 2:** % Cell viability/metabolic activity of primary cortical cells following peptide treatment (MTT assay).

Peptides. no.	Abbreviation	Concentration μM	Percent rate of proliferation with S.E.M	Significance
**1**	**LP** (LSAVTPG)	50	70.1 ± 1.23	0.001
		100	74.4 ± 3.22	0.001
**2**	**P6F** (LSAVT**F**G)	50	99.8 ± 1.64	1.000
		100	82.6 ± 2.18	0.007
**3**	**P6F.DMA** (LSAVTFG.DMA)	50	95.8 ± 3.16	1.000
		100	101.5 ± 1.39	1.000
**4**	L1 **A** ( **A** SAVTFG)	50	79.7 ± 3.03	0.001
		100	60.1 ± 3.83	0.001
**5**	L1 **A** .DMA **(A** SAVTFG.DMA)	50	94.7 ± 2.10	0.991
		100	73.5 ± 0.66	0.001
**6**	S2 **A** (L **A** AVTFG)	50	53.3 ± 3.40	0.001
		100	61.1 ± 2.06	0.001
**7**	S2 **A** .DMA (L **A** AVTFG.DMA)	50	84.2 ± 1.82	0.020
		100	84.3 ± 3.29	0.022
**8**	V4 **A** (LSA **A** TFG)	50	86.9 ± 0.54	0.096
		100	64.5 ± 2.11	0.001
**9**	T5 **A** (LSAV **A** FG)	50	90.3 ± 4.82	0.425
		100	79.7 ± 2.99	0.001
**10**	F6 **A** (LSAVT **A** G)	50	80.4 ± 4.55	0.002
		100	69.9 ± 1.12	0.001
**11**	G7 **A** (LSAVTF **A** )	50	110.7 ± 0.73	0.299
		100	90.8 ± 2.33	0.504
**12**	**L1A** (ASAVTFG)	50	97.7 ± 2.02	0.935
		100	89.8 ± 2.02	0.036
**13**	**L1A.DMA** (ASAVTFG.DMA)	50	65.7 ± 3.92	0.001
		100	69.9 ± 4.80	0.001
**14**	**S2A** (LAAVTFG)	50	94.3 ± 1.24	0.349
		100	84.3 ± 1.34	0.002
**15**	**S2A.DMA** (LAAVTFG.DMA)	50	75.8 ± 0.75	0.001
		100	72.5 ± 7.78	0.001
**16**	**V4A.DMA** (LSAATFG.DMA)	50	74.1 ± 3.75	0.001
		100	65.1 ± 3.52	0.001
**17**	**T5A.DMA** (LSAVAFG.DMA)	50	75.9 ± 3.25	0.001
		100	65.9 ± 2.21	0.001
**18**	**F6A.DMA** (LSAVTAG.DMA)	50	57.2 ± 3.38	0.001
		100	47.0 ± 1.83	0.001
**19**	**G7A.DMA** (LSAVT**F**A.DMA)	50	78.6 ± 4.73	0.001
		100	52.9 ± 4.42	0.001

*Note:* Values represent percentage metabolic activity relative to untreated control (set at 100%) as measured by MTT assay, reflecting overall cellular metabolic activity of a mixed culture; reductions may arise from neuronal differentiation and depletion of highly metabolically active fibroblast‐like cells rather than direct cytotoxicity.

Dunnett's post hoc test of one‐way ANOVA was employed to measure the significance difference between the control and test compound percent rate of proliferation. The most active peptides in terms of inducing prominent morphological differentiation into neuronal‐like cells, that is, **13** and **15** were further tested in OGD/R and Hypoxia/R Insult.

### 3.4 Neural Differentiation Activity of Peptides **13** and **15** Against OGD/R and Hypoxia/R Insult

**FIGURE 2:**
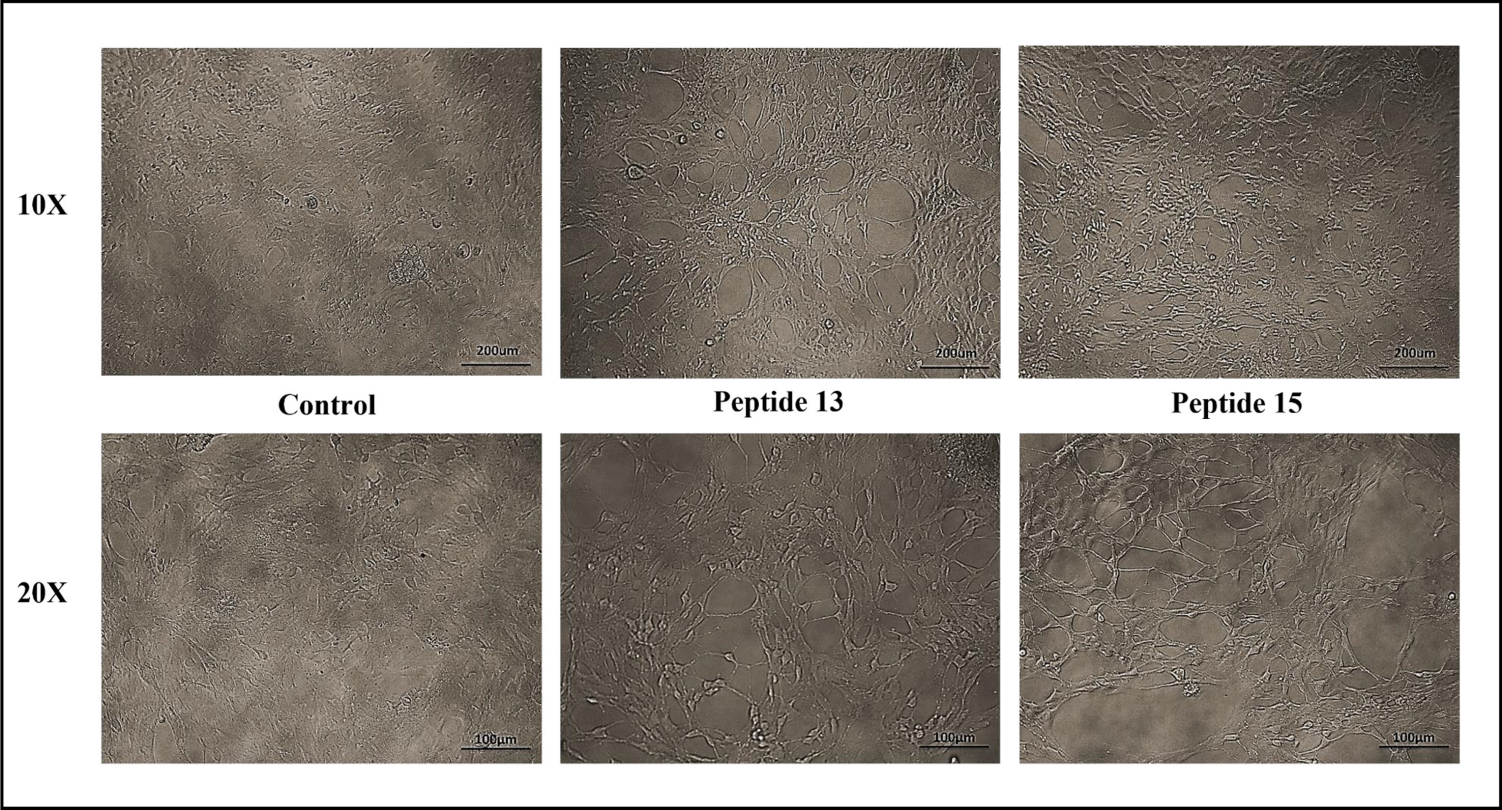
Phase contrast microscopic images displaying the differentiation of cortical cells by neuroprotective compounds. The cortical cells were treated with 100 μM concentration of peptides L1A.DMA (**13**) and S2A.DMA (**15**) for 48‐h duration. Following treatment, a prominent neuronal differentiation including elongated neurite‐like processes and neuronal morphology can be clearly seen in the treated cells. Images are displayed at 10× and 20× magnification. Scale bars = 100 μm. In the present study, our conclusions are primarily supported by qualitative morphological observations, including clear neurite extension and the emergence of neuron‐like phenotypes following peptide treatment. The observed morphological changes were constant and reproducible across independent experiments.

The alterations in the cell appearance and viability of cells are considered as a measure of cell damage assessment. After 21 h of reoxygenation phase in both the OGD and hypoxia insulted cells, the cells of each group were analyzed for the change in their appearance. The phase contrast microscopic photomicrographs clearly show that the cells of the OGD/R and hypoxia/R exposed group have broken neuronal connections, dispersive neuronal linings, and the cluster of dead cells. However, the peptides L1A.DMA (**13**) and S2A.DMA (**15**) at the 100 μM concentration demonstrated most promising results with respect to the neuronal differentiation potential effect on primary cortical cells (Figure 2).

**FIGURE 3:**
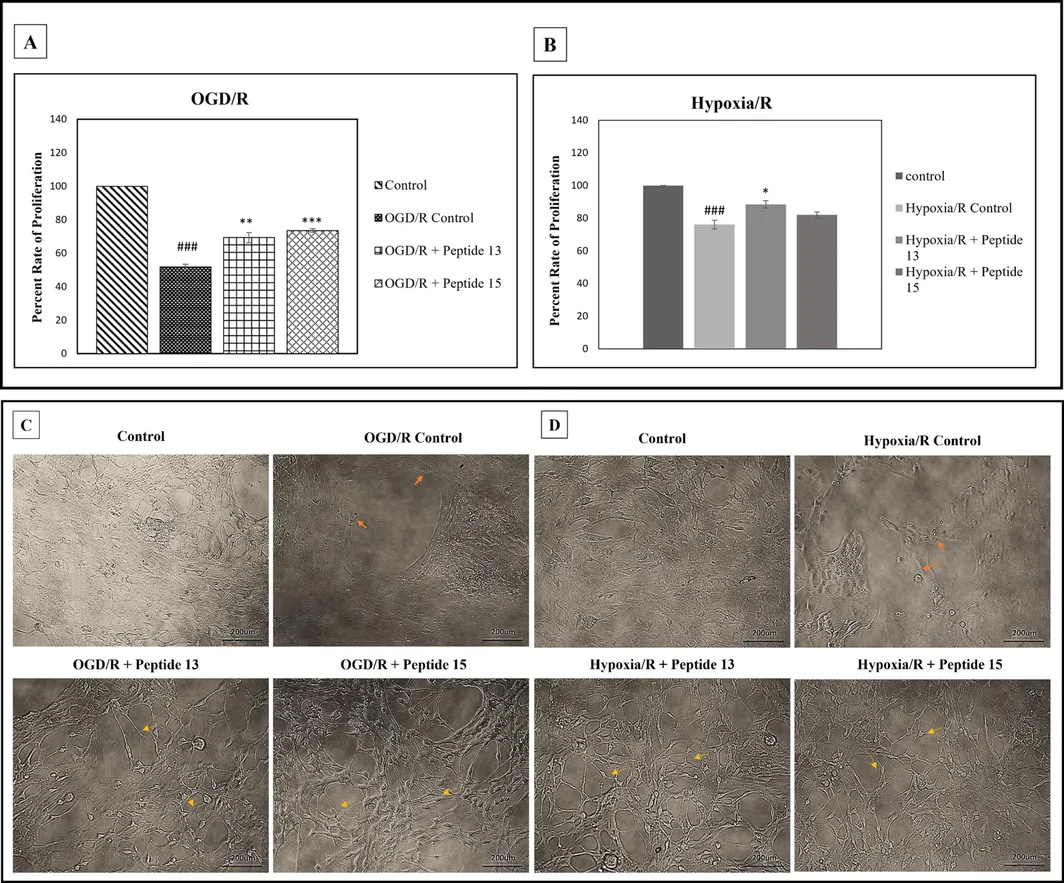
Neuroprotective activity demonstrated by peptides L1A.DMA (**13**) and S2A.DMA (**15**) against the cellular injury induced by OGD and hypoxia insult. (A, B) Bar graph displaying the percent rate of proliferation exhibited by peptides L1A.DMA (**13**) and S2A.DMA (**15**) pretreatment in comparison to the OGD/R and hypoxia/R groups. The level of significance is displayed as^*###*^ *p* ≤ 0.001 vs. control group; *p ≤ 0.05, ** *p* ≤ 0.01, and ***p ≤ 0.001 versus OGD/R and hypoxia/R groups. (**C**) The phase contrast images showing the preventive effect of peptides L1A.DMA (**13**) and S2A.DMA (**15**) against OGD/R insult at 20× magnification. (D) Photomicrographs of cortical cells pretreated with test compounds remarkably protected the cells against the detrimental stimuli of hypoxia insult. The red arrow shows cell death while the yellow ones are indicating signs of neuronal differentiation. Scale bars = 100 μm.

After morphological observation, the MTT assay was performed to determine the growth supporting activity (cell proliferation) possessed by the peptides in response to the ischemic insult. The MTT results displayed a significant decline in the percent rate of proliferation in both OGD/R and hypoxia/R exposed cells in comparison to the normal untreated control group (*p* ≤ 0.001). In comparison to the OGD/R group, the peptides L1A.DMA (**13**) and S2A.DMA (**15**) displayed a significant increase in the percent rate of proliferation (*p* ≤ 0.01, *p* ≤ 0.001, respectively) (Figure 3A). Additionally, both compounds showed growth supporting activity as compared to the hypoxia/R group but the results were only significant in peptide L1A.DMA (**13**) pretreated group (*p* ≤ 0.05) (Figure 3B). The measurement of significance among different groups was assessed by ANOVA with Bonferroni's post hoc test. An interesting observation was noted that despite a decrease in the rate of proliferation of the cells treated with peptides L1A.DMA (**13**) and S2A.DMA (**15**), the treated group showed strong neuronal connections and intact cellular linings, and synaptic connections after 21 h of reoxygenation phase in response to the detrimental stimuli of OGD (Figure 3C) and hypoxia (Figure 3D).

## 4 Discussion

**FIGURE 4:**
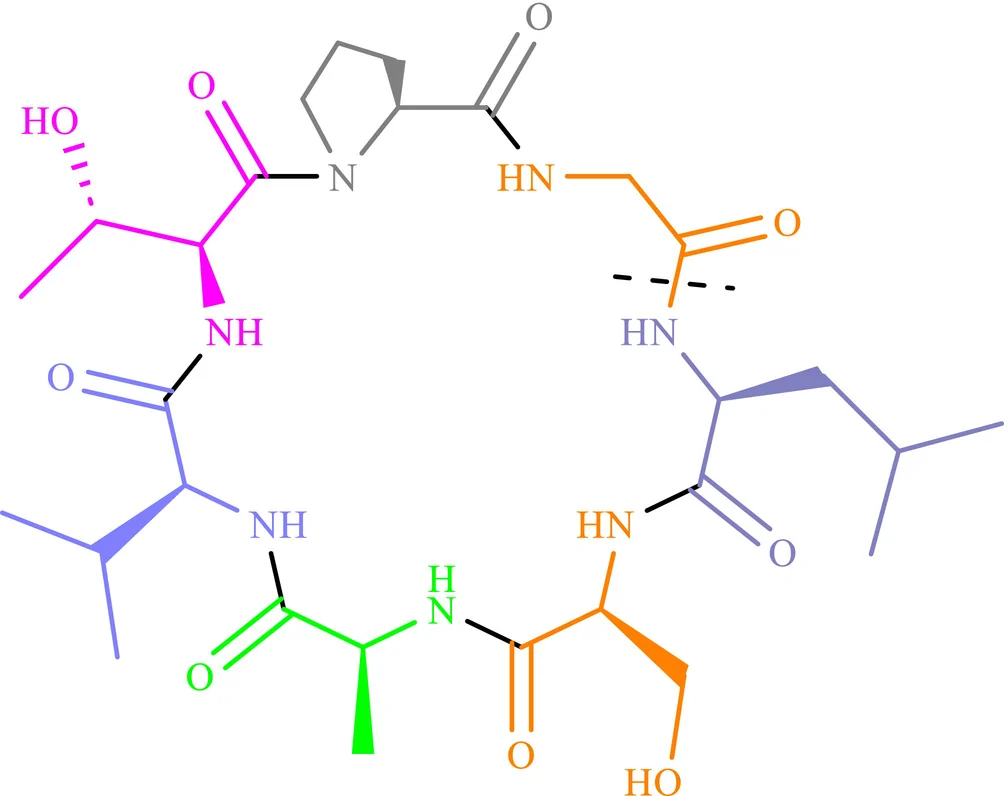
Selected starting position for linear precursor synthesis of *annomuricatin* F.

**FIGURE 5:**
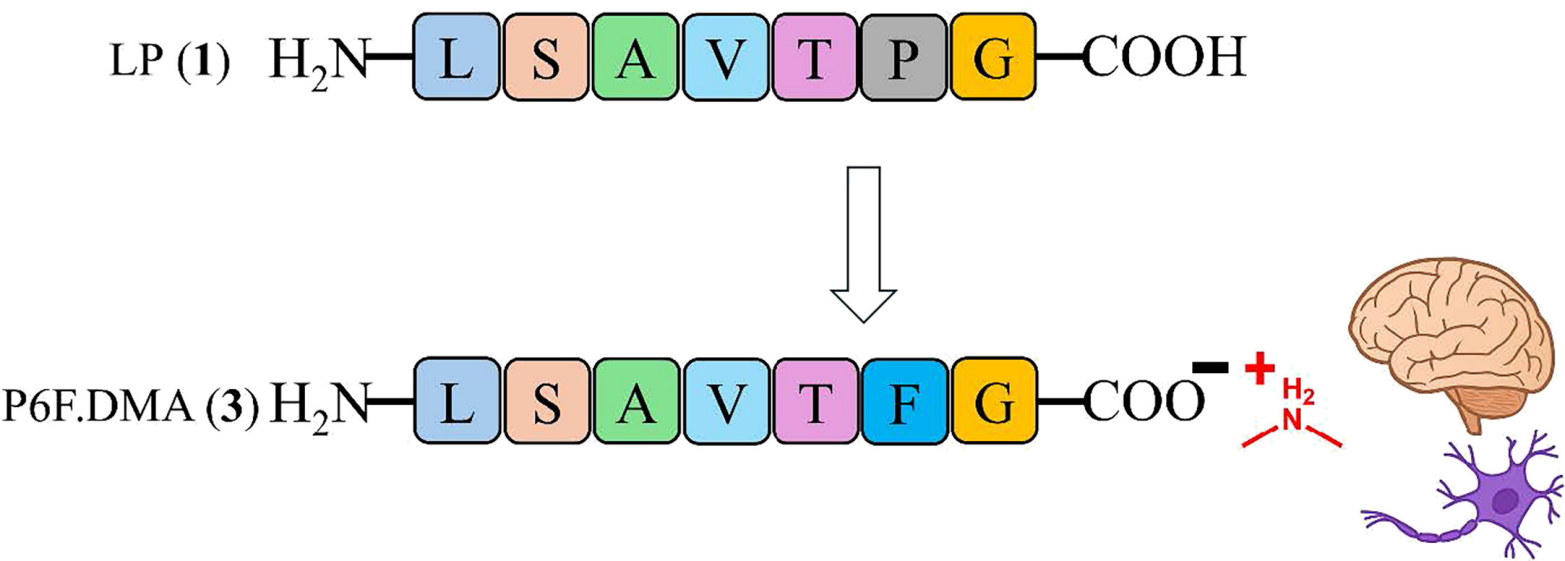
Modification of the linear precursor of *annumuricatin* F with Phe.

A heptapeptide *annomuricatin* F sequence cyclo‐(Leu‐Ser‐Ala‐Val‐Thr‐Pro‐Gly) was reported from the medicinal soursop plant, exhibiting significant cytotoxic and anti‐inflammatory activities [24]. In the current study, the linear precursor (LP) of *annomuricatin* F (Figure 4) was synthesized and fully characterized. Initial attempts to prepare cyclic peptide from LP were not successful. Therefore, some linear peptide analogs of LP were prepared. Among them, the peptide **2** was designed to synthesize by substituting the proline residue at position 6 of peptide LP (**1**) with phenylalanine (Figure 5), which led to the synthesis of peptide‐associated salt P6F.DMA (**3**). We subsequently resynthesized the peptide in its salt‐free form P6F (**2**) by changing the conditions used in final step of peptide purification. These peptides were evaluated for their effect on neuronal cell growth using MTT assay.

The peptide P6F.DMA (**3**) exhibited neuronal and nonneuronal cell growth activity (Table 2). To enhance the potency of peptide P6F.DMA (**3**), we performed L‐ and D‐alanine scanning to identify the amino acids essential for its activity. Sixteen derivatives (**4**–**19**) of peptide P6F.DMA (**3**) were prepared, each containing a single amino acid replaced with either L‐ or D‐alanine and including salt and nonsalt forms.

The prominent neuronal differentiation was observed when Leu and Ser were replaced in peptide **2** with L‐Ala yielding peptide **13** and peptide **15**, respectively, and the same unsalted peptides (Peptide **12** and Peptide **14**) demonstrated no prominent effect on the growth of the neuronal and nonneuronal cell population in the culture (Figure S120). However, the differentiation of the NPCs/NSCs was minimal (based on the morphological evaluation). When D‐alanine was inserted into the same location, the resulting peptides **5** and **7,** respectively, **e**xhibited no effect on cellular growth and their salt‐free counterparts, Peptides **4** and **6**, likewise exhibited no activity (Figure S121). While the rest of the residue's substitutions in peptide **2** with D‐ or L‐Ala exhibited variable activities.

Based on the MTT results, the experiment was further set to analyze the prominent neural differentiating activities exhibited by peptides Peptide **13** and **15,** against the in vitro OGD/R ischemia‐like injury, a widely accepted model that mimics the pathophysiological events of cerebral ischemia. The OGD/R produces critical features of ischemic pathology, thereby providing a reliable system for mechanistic study of neuroprotective agents. It was interesting to see that the treatment with the test compound demonstrated a marked attenuation of OGD/R‐induced cytotoxicity, suggesting its ability to preserve cellular viability under hypoxic–ischemic conditions. These protective effects may be attributed to the compound's potential to stabilize mitochondrial integrity, reduce oxidative stress, or modulate apoptotic signaling cascades. Notably, the observed neuroprotection is in line with previous reports highlighting the efficacy of small molecules and peptides in mitigating ischemia‐related injury. Collectively, our findings support the therapeutic potential of the test compound in ischemic pathologies and warrant further investigation into its precise molecular targets as well as validation in in vivo stroke models.

The rationale for studying these peptides in the ischemia‐like pathology was based on the fact that current pharmacological therapies for ischemic stroke remain insufficiently effective, emphasizing the need for new approaches to identify therapeutic targets and potential neuroprotective agents. Over the past few years, peptides have drawn significant attention in stroke research due to their ability to regulate the pathological processes provoked by reduced cerebral blood flow. Goldberg and Choi initially developed the OGD/R protocol by providing ischemic insult to the neocortical cultures at different time intervals that result in cellular injury [29]. This method is commonly used to mimic the ischemic insult in vitro settings [30]. Hypoxia is another critical factor for the survival and cellular death during ischemic insult. A cascade of pathological events is initiated in hypoxic events that lead to tissue necrosis due to lack of oxygen, excitation, and overexpression of inflammatory cytokines such as TNF‐α and IL‐1β [31, 32]. The results demonstrated significant decline in the viability of cells in OGD/R and hypoxia/R disease control groups in comparison to the control cells, which is consistent with the reported data. However, this cellular injury was prevented by the pretreatment with peptides L1A.DMA (**13**) and S2A.DMA (**15**) and displayed increase in percent rate of proliferation.

Here, it is to be noted that the primary cortical cultures are heterogeneous, containing neurons, NPCs, and population of proliferative nonneuronal cells (e.g., fibroblast‐like/glial cells). The peptides under investigation are hypothesized to promote neuronal differentiation rather than true proliferation of mature neurons. Over time, the following biological changes occur in cultured cortical cells:

- NPCs undergo differentiation into mature neuronal cell types under the influence of the test peptides.
- The proliferative nonneuronal cell population gradually declines.
- Differentiated neurons exhibit lower metabolic activity per cell and reduced mitochondrial turnover compared to actively dividing cells.

Because the MTT assay measures mitochondrial metabolic activity (NAD(P)H‐dependent oxidoreductase activity) rather than direct cell number, the observed values (70%–110%) in the Table 2, reflect relative metabolic activity compared to untreated control cultures, not an absolute measure of proliferation. Therefore, values around 100%–110% indicate maintained or slightly enhanced metabolic activity/viability. Whereas values around 70% do not necessarily indicate 30% cell death but may reflect reduced metabolic rate associated with cell differentiation, decreased contribution from proliferating nonneuronal cells, and a shift in overall culture composition toward mature neuronal cells with lower MTT reduction capacity. Here, it is clarifying that neuronal differentiation, and neuroprotective effects are supported by morphological observations including clear neurite extension and the emergence of neuron‐like phenotypes following peptide treatment, and complementary assays, rather than relying solely on MTT data. The morphological evaluation of all groups by phase contrast microscopy confirmed the aforementioned results of the MTT assay. These results verify that the peptides L1A.DMA (**13**) and S2A.DMA (**15**) pretreatment protected the cells from the detrimental effects of ischemia and hypoxia and exhibited neuroprotective effects.

Here, it is worth mentioning that the observed neurogenic effects characterized by reduced MTT absorbance alongside morphological features consistent with neuronal differentiation were primarily apparent at comparatively higher peptide concentrations, that is, 50–100 μM. While these findings support a potential role of the peptides in promoting neuronal differentiation, further studies employing a wider and lower concentration range, can be carried out to better define their potency and specificity.

## 5 Conclusions

This work describes the synthesis and biological evaluation of a heptapeptide derived from the medicinal *soursop* plant, alongside targeted amino acid substitutions that led to serendipitous discovery of peptides L1A.DMA (**13**) and S2A.DMA (**15**), which demonstrated significant protective or resilience‐enhancing effects against OGD/R‐ and hypoxia/R‐induced cellular stress as evidenced by cell viability/metabolic activity and supportive morphological changes, thus highlighting their promise as lead structures for the development of peptide‐based neuroprotective therapeutics.

## Conflicts of Interest

The authors declare no conflicts of interest.

## Supporting information




**Figure S1:** HR‐FAB‐MS (positive mode) of peptide LP (**1**).
**Figure S2:** ^1^H‐NMR (600 MHz) spectra of peptide LP (**1**) in DMSO‐*d6*.
**Figure S3:** HSQC correlations of peptide LP (**1**).
**Figure S4:** COSY correlations of peptide LP (**1**).
**Figure S5:** BB of peptide LP (**1**).
**Figure S6:** HR‐FAB‐MS (positive mode) of peptide P6F (**2**).
**Figure S7:** ^1^H‐NMR (600 MHz) spectra of peptide P6F (**2**) in DMSO‐*d6*.
**Figure S8:** HSQC correlations of peptide P6F (**2**).
**Figure S9:** COSY correlations of peptide P6F (**2**).
**Figure S10:** BB of peptide P6F (**2**).
**Figure S11:** HR‐FAB‐MS (positive mode) of peptide P6F.DMA (**3**).
**Figure S12:** ^1^H‐NMR (600 MHz) spectra of peptide P6F.DMA (**3**) in DMSO‐*d6*.
**Figure S13:** HSQC correlations of peptide P6F.DMA (**3**).
**Figure S14:** COSY correlations of peptide P6F.DMA (**3**).
**Figure S15:** HMBC correlations of peptide P6F.DMA (**3**).
**Figure S16:** DEPT 135 of peptide P6F.DMA (**3**).
**Figure S17:** DEPT 90 of peptide P6F.DMA (**3**).
**Figure S18:** BB of peptide P6F.DMA (**3**).
**Figure S19:** DOSY spectra of peptide P6F.DMA (**3**).
**Figure S20:** HR‐FAB‐MS (positive mode) of peptide L1 **A** (**4**).
**Figure S21:** ^1^H‐NMR (600 MHz) spectra of peptide L1 **A** (**4**) in DMSO‐*d6*.
**Figure S22:** HSQC correlations of peptide L1 **A** (**4**).
**Figure S23:** COSY correlations of peptide L1 **A** (**4**).
**Figure S24:** BB of peptide L1 **A** (**4**).
**Figure S25:** HR‐FAB‐MS (positive mode) of peptide L1 **A** .DMA (**5**).
**Figure S26:** ^1^H‐NMR (600 MHz) spectra of peptide L1 **A** .DMA (**5**) in DMSO‐*d6*.
**Figure S27:** HSQC correlations of peptide L1 **A** .DMA (**5**).
**Figure S28:** COSY correlations of peptide L1 **A** .DMA (**5**).
**Figure S29:** BB of peptide L1 **A** .DMA (**5**).
**Figure S30:** DOSY spectra of peptide L1 **A** .DMA (**5**).
**Figure S31:** HR‐FAB‐MS (positive mode) of peptide S2 **A** (**6**).
**Figure S32:** ^1^H‐NMR (600 MHz) spectra of peptide S2 **A** (**6**) in DMSO‐*d6*.
**Figure S33:** HSQC correlations of peptide S2 **A** (**6**).
**Figure S34:** COSY correlations of peptide S2 **A** (**6**).
**Figure S35:** BB of peptide S2 **A** (**6**).
**Figure S36:** HR‐FAB‐MS (positive mode) of peptide S2 **A** .DMA (**7**).
**Figure S37:** ^1^H‐NMR (600 MHz) spectra of peptide S2 **A** .DMA (**7**) in DMSO‐*d6*.
**Figure S38:** HSQC correlations of peptide S2 **A** .DMA (**7**).
**Figure S39:** COSY correlations of peptide S2 **A** .DMA (**7**).
**Figure S40:** BB of peptide S2 **A** .DMA (**7**).
**Figure S41:** DOSY spectra of peptide S2 **A** .DMA (**7**).
**Figure S42:** HR‐FAB‐MS (positive mode) of peptide V4 **A** (**8**).
**Figure S43:** ^1^H‐NMR (600 MHz) spectra of peptide V4 **A** (**8**) in DMSO‐*d6*.
**Figure S44:** HSQC correlations of peptide V4 **A** (**8**).
**Figure S45:** COSY correlations of peptide V4 **A** (**8**).
**Figure 46:** BB of peptide V4 **A** (**8**).
**Figure S47:** HR‐FAB‐MS (positive mode) of peptide T5 **A** (**9**).
**Figure S48:** ^1^H‐NMR (600 MHz) spectra of peptide T5 **A** (**9**) in DMSO‐*d6*.
**Figure S49:** HSQC correlations of peptide T5 **A** (**9**).
**Figure S50:** COSY correlations of peptide T5 **A** (**9**).
**Figure S51:** BB of peptide T5 **A** (**9**).
**Figure S52:** HR‐FAB‐MS (positive mode) of peptide F6 **A** (**10**).
**Figure S53:** ^1^H‐NMR (600 MHz) spectra of peptide F6 **A** (**10**) in DMSO‐*d6*.
**Figure S54:** HSQC correlations of peptide F6 **A** (**10**).
**Figure S55:** COSY correlations of peptide F6 **A** (**10**).
**Figure S56:** BB of peptide F6 **A** (**10**).
**Figure S57:** HR‐FAB‐MS (positive mode) of peptide G7 **A** (**11**).
**Figure S58:** ^1^H‐NMR (600 MHz) spectra of peptide G7 **A** (**11**) in DMSO‐*d6*.
**Figure S59:** HSQC correlations of peptide G7 **A** (**11**).
**Figure S60:** COSY correlations of peptide G7 **A** (**11**).
**Figure S61:** BB of peptide G7 **A** (**11**).
**Figure S62:** HR‐FAB‐MS (positive mode) of peptide L1A (**12**).
**Figure S63:** ^1^H‐NMR (600 MHz) spectra of peptide L1A (**12**) in DMSO‐*d6*.
**Figure S64:** HSQC correlations of peptide L1A (**12**).
**Figure S65:** COSY correlations of peptide L1A (**12**).
**Figure S66:** HMBC correlations of peptide L1A (**12**).
**Figure S67:** DEPT 135 of peptide L1A (**12**).
**Figure S68:** DEPT 90 of peptide L1A (**12**).
**Figure S69:** BB of peptide L1A (**12**).
**Figure S70:** HR‐FAB‐MS (positive mode) of peptide L1A.DMA (**13**).
**Figure S71:** ^1^H‐NMR (600 MHz) spectra of peptide L1A.DMA (**13**) in DMSO‐*d6*.
**Figure S72:** HSQC correlations of peptide L1A.DMA (**13**).
**Figure S73:** COSY correlations of peptide L1A.DMA (**13**).
**Figure S74:** HMBC correlations of peptide L1A.DMA (**13**).
**Figure S75:** DEPT 135 of peptide L1A.DMA (**13**).
**Figure S76:** DEPT 90 of peptide L1A.DMA (**13**).
**Figure S77:** BB of peptide L1A.DMA (**13**).
**Figure S78:** DOSY spectra of peptide L1A.DMA (**13**).
**Figure S79:** HR‐FAB‐MS (positive mode) of peptide S2A (**14**).
**Figure S80:** ^1^H‐NMR (600 MHz) spectra of peptide S2A (**14**) in DMSO‐*d6*.
**Figure S81:** HSQC correlations of peptide S2A (**14**).
**Figure S82:** COSY correlations of peptide S2A (**14**).
**Figure S83:** HMBC correlations of peptide S2A (**14**).
**Figure S84:** DEPT 135 of peptide S2A (**14**).
**Figure S85:** DEPT 90 of peptide S2A (**14**).
**Figure S86:** BB of peptide S2A (**14**).
**Figure S87:** HR‐FAB‐MS (positive mode) of peptide S2A.DMA (**15**).
**Figure S88:** ^1^H‐NMR (600 MHz) spectra of peptide S2A.DMA (**15**) in DMSO‐*d6*.
**Figure S89:** HSQC correlations of peptide S2A.DMA (**15**).
**Figure S90:** COSY correlations of peptide S2A.DMA (**15**).
**Figure S91:** HMBC correlations of peptide S2A.DMA (**15**).
**Figure S92:** DEPT 135 of peptide S2A.DMA (**15**).
**Figure S93:** DEPT 90 of peptide S2A.DMA (**15**).
**Figure S94:** BB of peptide S2A.DMA (**15**).
**Figure S95:** DOSY spectra of peptide S2A.DMA (**15**).
**Figure S96:** HR‐FAB‐MS (positive mode) of peptide V4A.DMA (**16**).
**Figure S97:** ^1^H‐NMR (600 MHz) spectra of peptide V4A.DMA (**16**) in DMSO‐*d6*.
**Figure S98:** HSQC correlations of peptide V4A.DMA (**16**).
**Figure S99:** COSY correlations of peptide V4A.DMA (**16**).
**Figure S100:** BB of peptide V4A.DMA (**16**).
**Figure S101:** DOSY spectra of peptide V4A.DMA (**16**).
**Figure S102:** HR‐FAB‐MS (positive mode) of peptide T5A.DMA (**17**).
**Figure S103:** ^1^H‐NMR (600 MHz) spectra of peptide T5A.DMA (**17**) in DMSO‐*d6*.
**Figure S104:** HSQC correlations of peptide T5A.DMA (**17**).
**Figure S105:** COSY correlations of peptide T5A.DMA (**17**).
**Figure S106:** BB of peptide T5A.DMA (**17**).
**Figure S107:** DOSY spectra of peptide T5A.DMA (**17**).
**Figure S108:** HR‐FAB‐MS (positive mode) of peptide F6A.DMA (**18**).
**Figure S109:** ^1^H‐NMR (600 MHz) spectra of peptide F6A.DMA (**18**) in DMSO‐*d6*.
**Figure S110:** HSQC correlations of peptide F6A.DMA (**18**).
**Figure S111:** COSY correlations of peptide F6A.DMA (**18**).
**Figure S112:** BB of peptide F6A.DMA (**18**).
**Figure S113:** DOSY spectra of peptide F6A.DMA (**18**).
**Figure S114:** HR‐FAB‐MS (positive mode) of peptide G7A.DMA (**19**).
**Figure S115:** ^1^H‐NMR (600 MHz) spectra of peptide G7A.DMA (**19**) in DMSO‐*d6*.
**Figure S116:** HSQC correlations of peptide G7A.DMA (**19**).
**Figure S117:** COSY correlations of peptide G7A.DMA (**19**).
**Figure S118:** BB of peptide G7A.DMA (**19**).
**Figure S119:** DOSY spectra of peptide G7A.DMA (**19**).

## Data Availability

The data that support the findings of this study are available from the corresponding author upon reasonable request.
