# Supplementary material for: Serendipitous Discovery of Short Peptides Dimethylammonium Salt Promoting Neural Differentiation
Source: J Pept Sci. 2026 Aug 20;32(10):e70124. doi: 10.1002/psc.70124 (PMC13491000; doi:10.1002/psc.70124)
Supplement: Supplementary file 1 [file PSC-32-e70124-s001.docx]

**EXPERIMENTAL PROCEDURES**

**General experimental procedures**

Annomuricatin F linear precursor and its analogs were synthesized manually according to the standard Nα-Fmoc protocol on a Wang resin (1.4 mmol/g substitution). Peptide chains were elongated using repeated cycles of deprotection and acylation. Deprotection of Fmoc groups was conducted using a 20% 4-methyl piperidine solution in DMF (1 × 20 min). Coupling reactions were carried out using 3 eq. of protected amino acids (Fmoc-AA), Oxyma Pure, and DIC (1:1:1) dissolved in DMF. The ester bond formation was accelerated by DMAP in the first coupling. Peptide cleavage and side-chain deprotection were accomplished in 2 h through a one-step procedure using a mixture of TFA/H2O (95:5). Subsequently, the peptides were purified by puriFlash 5.125 Preparative reversed-phase high-performance liquid chromatography (RP-HPLC) with Interchim^®^ software. Purifications were carried out on a PFB15C18XS-250/212 C18 column (21.20 × 250 mm, 15-μm particle size, 100-Å pore size). UV detection at 214 nm was used, and the crude peptides were eluted with an isocratic 60% acetonitrile in deionized water at room temperature. The mobile phase flow rate was 4.0 mL/min. Eluent contained 0.1% (v/v) of TFA. Fractions were analyzed by ultra-performance liquid chromatography (UPLC) (Agilent 1260 Infinity Diode Array, C-4 reversed-phase analytical column, 5 μm, 150 4.6 mm), mobile phase: acetonitrile/water (60:40) in 0.08% TFA). LR-FAB and HR-FAB mass spectra were recorded on QSTAR XL MS/MS systems (Agilent, USA). Bruker Avance 600 and 800 MHz NMR spectrometer were used for NMR spectra. NMR data were collected at 25^0^C.

1.1 L-leucyl-L-seryl-L-alanyl-L-valyl-L-allothreonyl-L-prolylglycine (LP)

Peptide LP (**1**) was synthesized on Wang resin (0.5 g, 1.2 mmol/g). Overall yield: (350 mg, 70 %); t_R_: 2.79 min (ACN:H_2_O:TFA; 60:40:0.08); ^1^H NMR (*d_6_*-DMSO, 600 MHz): 0.78 (3H, *d, J=* 6.8 Hz, Val^4^ γ^1^-CH_3_), 0.80 (3H, *d, J=* 6.6 Hz, Val^4^ γ^2^-CH_3_), 0.87 (3H, *d, J=* 6.4 Hz, Leu^1^ δ^1^-CH_3_), 0.89 (3H, *d, J=* 6.4 Hz, Leu^1^ δ^2^-CH_3_), 1.09 (3H, *d, J=* 6.3 Hz, Thr^5^ γ -CH_3_), 1.20 (3H, *d, J=* 6.9 Hz, Ala^3^ β -CH_3_), 1.49 (1H, *m*, Leu^1^ *β*-CH_2_), 1.55 (1H, *m*, Leu^1^ *β*-CH_2_), 1.63 (1H, *m*, Leu^1^ *γ*-CH), 1.81 (2H, *m*, Pro^6^ *β*-CH_2,_ *γ*-CH_2_), 1.91 (2H, *m*, Pro^6^ *γ*-CH_2,_ Val^4^ *β*-CH), 2.01 (1H, *m*, Pro^6^ *β*-CH_2_), 3.58 (2H, *m*, Ser^2^ *β*-CH_2_), 3.76-3.64 (4H, *m*, Pro^6^ *δ*-CH_2,_ Gly^7^ *α*-CH_2_), 3.85-3.83 (2H, *m*, Leu^1^ *α*-CH_,_ Thr^5^ *β*-CH), 4.22 (1H, *dd*, *J=* 6.6 Hz, 8.8 Hz, Val^4^ *α*-CH), 4.34-4.31 (2H, *m*, Pro^6^ *α*-CH_,_ Ala^3^ *α*-CH), 4.42-4.38 (2H, *m*, Ser^2^ *α*-CH_,_ Thr^5^ *α*-CH), 4.79 (1H, *br. s*, Thr^5^ OH), 5.10 (1H, *br. s*, Ser^2^ OH), 7.83 (1H, *d, J=* 9.0 Hz, Val^4^ NH), 7.92 (1H, *d, J=* 7.1 Hz, Thr^5^ NH), 8.08-8.06 (2H, *m*, Gly^7^ NH_,_ Leu^1^ NH), 8.16 (1H, *d, J=* 7.2 Hz, Ala^5^ NH), 8.68 (1H, *d, J=* 7.8 Hz, Ser^2^ NH); ^13^C-NMR (*d_6_*-DMSO, 150 MHz): *δ* ppm 17.9, 18.0, 19.1, 19.2, 21.7, 22.6, 23.4, 24.3, 29.2, 30.7, 40.1, 40.4, 47.2, 48.3, 50.7, 54.8, 56.3, 57.2, 59.2, 61.7, 66.7, 168.7, 168.9, 169.1, 170.7, 171.0, 171.7, 171.9; HR-FAB-MS (M + H)^+^: Calculated for [C_28_H_49_N_7_O_10_ + H]^+^: 644.3619; found: 644.3625.

1.2 L-leucyl-L-seryl-L-alanyl-L-valyl-L-allothreonyl-L-phenylalanylglycine (P6F)

Peptide P6F (**2**) was synthesized on Wang resin (0.5 g, 1.2 mmol/g). Overall yield: (460 mg, 92 %); t_R_: 2.90 min (ACN:H_2_O:TFA; 60:40:0.08); ^1^H NMR (*d_6_*-DMSO, 600 MHz): 0.76 (3H, *d, J=* 7.2 Hz, Val^4^ γ^1^-CH_3_), 0.78 (3H, *d, J=* 6.6 Hz, Val^4^ γ^2^-CH_3_), 0.87 (3H, *d, J=* 6.6 Hz, Leu^1^ δ^1^-CH_3_), 0.89 (3H, *d, J=* 6.0 Hz, Leu^1^ δ^2^-CH_3_), 0.95 (3H, *d, J=* 6.0 Hz, Thr^5^ γ -CH_3_), 1.20 (3H, *d, J=* 7.2 Hz, Ala^3^ β -CH_3_), 1.45 (1H, *m*, Leu^1^ *β*-CH_2_), 1.54 (1H, *m*, Leu^1^ *β*-CH_2_), 1.64 (1H, *m*, Leu^1^ *γ*-CH), 1.95 (1H, *m*, Val^4^ *β*-CH), 2.80 (1H, *dd*, *J=* 9.0, 14.4 Hz, Phe^6^ *β*-CH_2_), 3.06 (1H, *dd*, *J=* 4.8, 14.4 Hz, Phe^6^ *β*-CH_2_), 3.58 (2H, *m*, Ser^2^ *β*-CH_2_), 3.68 (2H, *m*, Gly^7^ *α*-CH_2_), 3.76 (1H, *t*, *J=* 6.7 Hz, Leu^1^ *α*-CH), 3.91 (1H, *m*, Thr^5^ *β*-CH), 4.18-4.21 (2H, *m*, Val^4^ *α*-CH, Thr^5^ *α*-CH), 4.33 (1H, *m*, Ala^3^ *α*-CH), 4.40 (1H, *m*, Ser^2^ *α*-CH), 4.55 (1H, *m*, Phe^6^ *α*-CH), 5.02 (1H, *br. s*, Thr^5^ OH), 5.04 (1H, *br. s*, Ser^2^ OH), 7.14 (1H, *m*, Phe^6^ CH_Ar_), 7.21 (4H, *m*, Phe^6^ CH_Ar_), 7.70 (1H, *d, J=* 7.8 Hz, Thr^5^ NH), 7.86 (1H, *d, J=* 9.0 Hz, Val^4^ NH), 7.88 (1H, *d, J=* 7.8 Hz, Phe^6^ NH), 8.15-8.18 (2H, *m*, Ala^3^ NH, Gly^7^ NH), 8.68 (1H, *d, J=* 7.8 Hz, Ser^2^ NH); ^13^C-NMR (*d_6_*-DMSO, 150 MHz): *δ* ppm 17.8, 18.0, 19.1, 21.8, 22.7, 23.4, 30.4, 37.4, 40.2, 40.6, 48.3, 50.7, 53.5, 54.8, 57.5, 57.7, 61.7, 66.5, 126.2, 128.0, 129.1, 137.5, 169.0, 169.1, 169.3, 170.8, 170.9, 171.1, 172.0; HR-FAB-MS (M + H)^+^: Calculated for [C_32_H_51_N_7_O_10_ + H]^+^: 694.3776; found: 694.3811.

1.3 Dimethylammonium L-leucyl-L-seryl-L-alanyl-L-valyl-L-allothreonyl-L-phenylalanylglycinate (P6F.DMA)

Peptide P6F.DMA (**3**) was synthesized on Wang resin (0.5 g, 1.2 mmol/g). Overall yield: (435 mg, 87 %); t_R_: 2.84 min (ACN:H_2_O:TFA; 60:40:0.08); ^1^H NMR (*d_6_*-DMSO, 600 MHz): 0.76 (3H, *d, J=* 6.9 Hz, Val^4^ γ^1^-CH_3_), 0.78 (3H, *d, J=* 6.7 Hz, Val^4^ γ^2^-CH_3_), 0.86 (3H, *d, J=* 6.4 Hz, Leu^1^ δ^1^-CH_3_), 0.88 (3H, *d, J=* 6.5 Hz, Leu^1^ δ^2^-CH_3_), 0.95 (3H, *d, J=* 6.3 Hz, Thr^5^ γ -CH_3_), 1.20 (3H, *d, J=* 7.0 Hz, Ala^3^ β -CH_3_), 1.45 (1H, *m*, Leu^1^ *β*-CH_2_), 1.54 (1H, *m*, Leu^1^ *β*-CH_2_), 1.64 (1H, *m*, Leu^1^ *γ*-CH), 1.95 (1H, *m*, Val^4^ *β*-CH), 2.53 (1H, *s*, dMA CH_3_), 2.81 (1H, *dd*, *J=* 9.1, 14.0 Hz, Phe^6^ *β*-CH_2_), 3.05 (1H, *dd*, *J=* 4.6, 14.1 Hz, Phe^6^ *β*-CH_2_), 3.58 (2H, *m*, Ser^2^ *β*-CH_2_), 3.68 (2H, *m*, Gly^7^ *α*-CH_2_), 3.76 (1H, *t*, *J=* 6.7 Hz, Leu^1^ *α*-CH), 3.93 (1H, *m*, Thr^5^ *β*-CH), 4.18-4.20 (2H, *m*, Val^4^ *α*-CH, Thr^5^ *α*-CH), 4.32 (1H, *m*, Ala^3^ *α*-CH), 4.39 (1H, *m*, Ser^2^ *α*-CH), 4.53 (1H, *m*, Phe^6^ *α*-CH), 5.04 (2H, *br. s*, Ser^2^ OH, Thr^5^ OH), 7.15 (1H, *m*, Phe^6^ CH_Ar_), 7.21 (4H, *m*, Phe^6^ CH_Ar_), 7.67 (1H, *d, J=* 8.2 Hz, Thr^5^ NH), 7.85 (1H, *d, J=* 8.8 Hz, Val^4^ NH), 7.92 (1H, *d, J=* 8.1 Hz, Phe^6^ NH), 8.15-8.18 (2H, *m*, Ala^3^ NH, Gly^7^ NH), 8.63 (1H, *d, J=* 6.5 Hz, Ser^2^ NH); ^13^C-NMR (*d_6_*-DMSO, 150 MHz): *δ* ppm 17.8, 18.0, 19.2, 21.8, 22.7, 23.5, 30.3, 34.3, 37.3, 40.6, 41.1, 48.3, 51.0, 53.6, 54.8, 57.6, 57.7, 61.8, 66.6, 126.2, 128.0, 129.1, 137.6, 169.2, 169.4, 169.8, 170.8, 170.8, 171.1, 172.1; HR-FAB-MS (M + H)^+^: Calculated for [C_32_H_51_N_7_O_10_ + H]^+^: 694.3776; found: 694.3789

1.4 D-alanyl-L-seryl-L-alanyl-L-valyl-L-allothreonyl-L-phenylalanylglycine (L1**A**)

Peptide L1**A** (**4**) was synthesized on Wang resin (0.5 g, 1.2 mmol/g). Overall yield: (445 mg, 89 %); t_R_: 2.87 min (ACN:H_2_O:TFA; 60:40:0.08); ^1^H NMR (*d_6_*-DMSO, 600 MHz): 0.77 (3H, *d, J=* 6.6 Hz, Val^4^ γ^1^-CH_3_), 0.79 (3H, *d, J=* 6.6 Hz, Val^4^ γ^2^-CH_3_), 0.95 (3H, *d, J=* 6.6 Hz, Thr^5^ γ -CH_3_), 1.20 (3H, *d, J=* 7.2 Hz, Ala^3^ β -CH_3_), 1.33 (3H, *d, J=* 7.2 Hz, Ala^1^ β -CH_3_), 1.94 (1H, *m*, Val^4^ *β*-CH), 2.81 (1H, *dd*, *J=* 9.0, 13.8 Hz, Phe^6^ *β*-CH_2_), 3.05 (1H, *dd*, *J=* 4.2, 13.8 Hz, Phe^6^ *β*-CH_2_), 3.54 (2H, *m*, Ser^2^ *β*-CH_2_), 3.74 (2H, *t, J=* 6.0 Hz, Gly^7^ *α*-CH_2_), 3.89-3.91 (2H, *m*, Thr^5^ *β*-CH, Ala^1^ *α*-CH), 4.18-4.21 (2H, *m*, Val^4^ *α*-CH, Thr^5^ *α*-CH), 4.32 (1H, *m*, Ala^3^ *α*-CH), 4.44 (1H, *m*, Ser^2^ *α*-CH), 4.55 (1H, *m*, Phe^6^ *α*-CH), 5.02 (1H, *br. s*, Thr^5^ OH), 5.04 (1H, *br. s*, Ser^2^ OH), 7.15 (1H, *m*, Phe^6^ CH_Ar_), 7.21 (4H, *m*, Phe^6^ CH_Ar_), 7.71 (1H, *d, J=* 7.8 Hz, Thr^5^ NH), 7.78 (1H, *d, J=* 9.0 Hz, Val^4^ NH), 7.88 (1H, *d, J=* 8.4 Hz, Phe^6^ NH), 8.02 (2H, *m*, Ala^1^ NH_2_), 8.30 (1H, *d, J=* 7.2 Hz, Ala^3^ NH), 8.33 (1H, *t, J=* 5.4 Hz, Gly^7^ NH), 8.53 (1H, *d, J=* 7.8 Hz, Ser^2^ NH); ^13^C-NMR (*d_6_*-DMSO, 150 MHz): *δ* ppm 17.4, 17.8, 17.9, 19.1, 30.4, 37.4, 40.6, 48.1, 48.3, 53.5, 54.7, 57.5, 57.7, 61.9, 66.6, 126.2, 128.0, 129.1, 137.5, 169.3, 169.3, 169.4, 170.8, 170.9, 171.1, 172.0; HR-FAB-MS (M + H)^+^: Calculated for [C_29_H_45_N_7_O_10_ + H]^+^: 652.3306; found: 652.3328.

1.5 Dimethylammonium D-alanyl-L-seryl-L-alanyl-L-valyl-L-allothreonyl-L-phenylalanylglycinate (L1**A**.DMA)

Peptide L1**A**.DMA (**5**) was synthesized on Wang resin (0.5 g, 1.2 mmol/g). Overall yield: (425 mg, 85 %); t_R_: 2.69 min (ACN:H_2_O:TFA; 60:40:0.08); ^1^H NMR (*d_6_*-DMSO, 600 MHz): 0.77 (3H, *d, J=* 6.8 Hz, Val^4^ γ^1^-CH_3_), 0.78 (3H, *d, J=* 6.7 Hz, Val^4^ γ^2^-CH_3_), 0.95 (3H, *d, J=* 6.3 Hz, Thr^5^ γ -CH_3_), 1.21 (3H, *d, J=* 7.1 Hz, Ala^3^ *β*-CH_3_), 1.32 (3H, *d, J=* 6.9 Hz, Ala^1^ *β*-CH_3_), 1.94 (1H, *m*, Val^4^ *β*-CH), 2.53 (6H, *s*, dMA CH_3_), 2.82 (1H, *dd*, *J=* 9.0, 14.1 Hz, Phe^6^ *β*-CH_2_), 3.06 (1H, *dd*, *J=* 4.6, 14.1 Hz, Phe^6^ *β*-CH_2_), 3.55 (2H, *m*, Ser^2^ *β*-CH_2_), 3.72 (2H, *m*, Gly^7^ *α*-CH_2_), 3.87 (1H, *m*, Ala^1^ *α*-CH), 3.93 (1H, *m*, Thr^5^ *β*-CH) 4.18-4.21 (2H, *m*, Val^4^ *α*-CH, Thr^5^ *α*-CH), 4.32 (1H, *m*, Ala^3^ *α*-CH), 4.42 (1H, *m*, Ser^2^ *α*-CH), 4.55 (1H, *m*, Phe^6^ *α*-CH), 5.02 (1H, *br. s*, Thr^5^ OH), 5.04 (1H, *br. s*, Ser^2^ OH), 7.15 (1H, *m*, Phe^6^ CH_Ar_), 7.21 (4H, *m*, Phe^6^ CH_Ar_), 7.70 (1H, *d, J=* 8.2 Hz, Thr^5^ NH), 7.78 (1H, *d, J=* 8.7 Hz, Val^4^ NH), 7.90 (1H, *d, J=* 8.2 Hz, Phe^6^ NH), 8.28-8.31 (2H, *m*, Ala^3^ NH_,_ Gly^7^ NH), 8.54 (1H, *d, J=* 8.1 Hz, Ser^2^ NH); ^13^C-NMR (*d_6_*-DMSO, 150 MHz): *δ* ppm 17.5, 17.8, 17.9, 19.1, 30.3, 34.2, 37.4, 40.8, 48.1, 48.3, 53.6, 54.7, 57.5, 57.7, 61.9, 66.5, 126.2, 128.0, 129.1, 137.5, 169.3, 169.3, 169.6, 170.8, 171.0, 171.0, 172.0; HR-FAB-MS (M + H)^+^: Calculated for [C_29_H_45_N_7_O_10_ + H]^+^: 652.3306; found: 652.3335.

1.6 L-leucyl-D-alanyl-L-alanyl-L-valyl-L-allothreonyl-L-phenylalanylglycine (S2**A**)

Peptide S2**A** (**6**) was synthesized on Wang resin (0.5 g, 1.2 mmol/g). Overall yield: (435 mg, 87 %); t_R_: 2.97 min (ACN:H_2_O:TFA; 60:40:0.08); ^1^H NMR (*d_6_*-DMSO, 600 MHz): 0.80 (6H, *d, J=* 7.2 Hz, Val^4^ γ^1,2^-CH_3_), 0.87 (3H, *d, J=* 6.6 Hz, Leu^1^ δ^1^-CH_3_), 0.89 (3H, *d, J=* 6.0 Hz, Leu^1^ δ^2^-CH_3_), 0.95 (3H, *d, J=* 6.6 Hz, Thr^5^ γ-CH_3_), 1.18 (3H, *d, J=* 7.2 Hz, Ala^3^ *β*-CH_3_), 1.20 (3H, *d, J=* 6.6 Hz, Ala^2^ *β*-CH_3_), 1.52 (2H, *m*, Leu^1^ *β*-CH_2_), 1.60 (1H, *m*, Leu^1^ *γ*-CH), 1.96 (1H, *m*, Val^4^ *β*-CH), 2.80 (1H, *dd*, *J=* 9.0, 10.8 Hz, Phe^6^ *β*-CH_2_), 3.03 (1H, *dd*, *J=* 4.8, 14.4 Hz, Phe^6^ *β*-CH_2_), 3.74 (2H, *m*, Gly^7^ *α*-CH_2_), 3.84 (1H, *m*, Leu^1^ *α*-CH), 3.92 (1H, *m*, Thr^5^ *β*-CH), 4.15 (1H, *m*, Val^4^ *α*-CH), 4.19 (1H, *m*, Thr^5^ *α*-CH), 4.35 (1H, *m*, Ala^3^ *α*-CH), 4.43 (1H, *m*, Ala^2^ *α*-CH), 4.55 (1H, *m*, Phe^6^ *α*-CH), 5.02 (1H, *br. s*, Thr^5^ OH), 7.14 (1H, *m*, Phe^6^ CH_Ar_), 7.21 (4H, *m*, Phe^6^ CH_Ar_), 7.64 (1H, *d, J=* 7.8 Hz, Thr^5^ NH), 7.89 (1H, *d, J=* 7.8 Hz, Phe^6^ NH), 7.91 (1H, *d, J=* 9.0 Hz, Val^4^ NH), 8.02 (2H, *m*, Leu^1^ NH_2_), 8.22 (1H, *m*, Ala^3^ NH), 8.35 (1H, *t, J=* 5.4 Hz, Gly^7^ NH), 8.75 (1H, *d, J=* 7.8 Hz, Ala^2^ NH); ^13^C-NMR (*d_6_*-DMSO, 150 MHz): *δ* ppm 18.1, 18.9, 19.0, 19.1, 19.2, 21.8, 22.6, 23.6, 30.1, 37.4, 40.2, 40.6, 48.0, 48.2, 50.7, 53.5, 57.5, 57.8, 66.5, 126.2, 128.0, 129.1, 137.5, 168.5, 169.3, 169.3, 170.9, 170.9, 171.1, 172.1; HR-FAB-MS (M + H)^+^: Calculated for [C_32_H_51_N_7_O_9_ + H]^+^: 678.3827; found: 678.3856.

1.7 Dimethylammonium L-leucyl-D-alanyl-L-alanyl-L-valyl-L-allothreonyl-L-phenylalanylglycinate (S2**A**.DMA)

Peptide S2**A**.DMA (**7**) was synthesized on Wang resin (0.5 g, 1.2 mmol/g). Overall yield: (445 mg, 89 %); t_R_: 2.76 min (ACN:H_2_O:TFA; 60:40:0.08); ^1^H NMR (*d_6_*-DMSO, 600 MHz): 0.76 (3H, *d, J=* 6.8 Hz, Val^4^ γ^1^-CH_3_), 0.78 (3H, *d, J=* 6.7 Hz, Val^4^ γ^2^-CH_3_), 0.86 (3H, *d, J=* 6.6 Hz, Leu^1^ δ^1^-CH_3_), 0.88 (3H, *d, J=* 6.5 Hz, Leu^1^ δ^2^-CH_3_), 0.95 (3H, *d, J=* 6.3 Hz, Thr^5^ *γ*-CH_3_), 1.18 (3H, *d, J=* 7.1 Hz, Ala^3^ *β*-CH_3_), 1.22 (3H, *d, J=* 7.1 Hz, Ala^2^ *β*-CH_3_), 1.40 (1H, *m*, Leu^1^ *β*-CH_2_), 1.49 (1H, *m*, Leu^1^ *β*-CH_2_), 1.64 (1H, *m*, Leu^1^ *γ*-CH), 1.96 (1H, *m*, Val^4^ *β*-CH), 2.53 (19H, *s*, dMA CH_3_), 2.81 (1H, *dd*, *J=* 9.0, 14.0 Hz, Phe^6^ *β*-CH_2_), 3.06 (1H, *dd*, *J=* 4.6, 14.1 Hz, Phe^6^ *β*-CH_2_), 3.61 (1H, *m*, Leu^1^ *α*-CH), 3.66 (2H, *m*, Gly^7^ *α*-CH_2_), 3.94 (1H, *m*, Thr^5^ *β*-CH), 4.18-4.20 (2H, *m*, Val^4^ *α*-CH, Thr^5^ *α*-CH), 4.30 (1H, *m*, Ala^3^ *α*-CH), 4.35 (1H, *m*, Ala^2^ *α*-CH), 4.52 (1H, *m*, Phe^6^ *α*-CH), 5.02 (1H, *br. s*, Thr^5^ OH), 7.15 (1H, *m*, Phe^6^ CH_Ar_), 7.21 (4H, *m*, Phe^6^ CH_Ar_), 7.64 (1H, *d, J=* 8.2 Hz, Val^4^ NH), 7.76 (1H, *d, J=* 8.7 Hz, Thr^5^ NH), 7.93 (1H, *d, J=* 8.1 Hz, Phe^6^ NH), 8.12 (1H, *d, J=* 7.4 Hz, Ala^3^ NH), 8.14 (1H, *br. s*, Gly^7^ NH), 8.52 (1H, *br. s*, Ala^2^ NH); ^13^C-NMR (*d_6_*-DMSO, 150 MHz): *δ* ppm 17.7, 17.9, 18.3, 19.1, 19.2, 21.8, 22.8, 23.6, 30.4, 34.4, 37.3, 41.1, 41.2, 47.9, 48.1, 51.2, 53.7, 57.4, 57.6, 66.6, 126.2, 128.0, 129.1, 137.6, 169.4, 170.7, 170.8, 170.9, 171.4, 171.9, 172.1; HR-FAB-MS (M + H)^+^: Calculated for [C_32_H_51_N_7_O_9_ + H]^+^: 678.3827; found: 678.3866.

1.8 L-leucyl-L-seryl-L-alanyl-D-alanyl-L-allothreonyl-L-phenylalanylglycine (V4**A**)

Peptide V4**A** (**8**) was synthesized on Wang resin (0.5 g, 1.2 mmol/g). Overall yield: (385 mg, 77 %); t_R_: 2.72 min (ACN:H_2_O:TFA; 60:40:0.08); ^1^H NMR (*d_6_*-DMSO, 600 MHz): 0.86 (3H, *d, J=* 6.6 Hz, Leu^1^ δ^1^-CH_3_), 0.88 (3H, *d, J=* 6.6 Hz, Leu^1^ δ^2^-CH_3_), 0.94 (3H, *d, J=* 6.6 Hz, Thr^5^ *γ*-CH_3_), 1.17 (3H, *d, J=* 6.6 Hz, Ala^4^ *β*-CH_3_), 1.21 (3H, *d, J=* 7.2 Hz, Ala^3^ *β*-CH_3_), 1.48 (1H, *m*, Leu^1^ *β*-CH_2_), 1.55 (1H, *m*, Leu^1^ *β*-CH_2_), 1.63 (1H, *m*, Leu^1^ *γ*-CH), 2.83 (1H, *dd*, *J=* 9.0, 13.8 Hz, Phe^6^ *β*-CH_2_), 3.07 (1H, *dd*, *J=* 4.2, 14.4 Hz, Phe^6^ *β*-CH_2_), 3.55 (1H, *dd, J=* 6.6, 10.2 Hz, Ser^2^ *β*-CH_2_), 3.65 (1H, *dd, J=* 6.6, 10.8 Hz, Ser^2^ *β*-CH_2_), 3.74 (2H, *m*, Gly^7^ *α*-CH_2_), 3.84 (1H, *m*, Leu^1^ *α*-CH), 3.94 (1H, *m*, Thr^5^ *β*-CH), 4.16 (1H, *dd*, *J=* 4.2, 8.4 Hz, Thr^5^ *α*-CH), 4.25 (1H, *m,*, Ala^3^ *α*-CH), 4.31 (1H, *m,* Ala^4^ *α*-CH), 4.41 (1H, *m,* Ser^2^ *α*-CH), 4.52 (1H, *m*, Phe^6^ *α*-CH), 5.02 (1H, *br. s*, Thr^5^ OH), 5.04 (1H, *br. s*, Ser^2^ OH), 7.15 (1H, *m*, Phe^6^ CH_Ar_), 7.21 (4H, *m*, Phe^6^ CH_Ar_), 7.79 (1H, *d, J=* 8.4 Hz, Thr^5^ NH), 7.94-7.97 (2H, *m*, Phe^6^ NH, Ala^4^ NH), 8.10 (2H, *br. s*, Leu^1^ NH_2_), 8.24 (1H, *t, J=* 5.4 Hz, Gly^7^ NH), 8.28 (1H, *d, J=* 7.2 Hz, Ala^3^ NH), 8.66 (1H, *d, J=* 7.8 Hz, Ser^2^ NH); ^13^C-NMR (*d_6_*-DMSO, 150 MHz): *δ* ppm 17.8, 18.2, 19.2, 21.8, 22.6, 23.5, 37.2, 40.2, 40.6, 48.4, 48.5, 50.7, 53.9, 54.6, 57.7, 61.7, 66.4, 126.2, 128.0, 129.1, 137.7, 169.0, 169.3, 169.5, 170.9, 171.3, 171.9, 172.4; HR-FAB-MS (M + H)^+^: Calculated for [C_30_H_47_N_7_O_10_ + H]^+^: 666.3463; found: 666.3457.

1.9 L-leucyl-L-seryl-L-alanyl-L-valyl-D-alanyl-L-phenylalanylglycine (T5**A**)

Peptide T5**A** (**9**) was synthesized on Wang resin (0.5 g, 1.2 mmol/g). Overall yield: (430 mg, 86 %); t_R_: 2.99 min (ACN:H_2_O:TFA; 60:40:0.08); ^1^H NMR (*d_6_*-DMSO, 600 MHz): 0.77 (3H, *d, J=* 6.6 Hz, Val^4^ γ^1^-CH_3_), 0.78 (3H, *d, J=* 6.6 Hz, Val^4^ γ^2^-CH_3_), 0.87 (3H, *d, J=* 6.6 Hz, Leu^1^ δ^1^-CH_3_), 0.88 (3H, *d, J=* 6.6 Hz, Leu^1^ δ^2^-CH_3_), 0.90 (3H, *d, J=* 7.2 Hz, Ala^5^ *β*-CH_3_), 1.19 (3H, *d, J=* 6.6 Hz, Ala^3^ *β*-CH_3_), 1.47 (1H, *m*, Leu^1^ *β*-CH_2_), 1.55 (1H, *m*, Leu^1^ *β*-CH_2_), 1.63 (1H, *m*, Leu^1^ *γ*-CH), 1.90 (1H, *m*, Val^4^ *β*-CH), 2.71 (1H, *m*, Phe^6^ *β*-CH_2_), 3.08 (1H, *dd*, *J=* 4.2, 13.8 Hz, Phe^6^ *β*-CH_2_), 3.58 (2H, *m*, Ser^2^ *β*-CH_2_), 3.77 (2H, *t, J=* 5.4 Hz, Gly^7^ *α*-CH_2_), 3.83 (1H, *m*, Leu^1^ *α*-CH), 4.15 (1H, *m*, Val^4^ *α*-CH), 4.25 (1H, *m*, Ala^5^ *α*-CH), 4.32 (1H, *m*, Ala^3^ *α*-CH), 4.40 (1H, *m*, Ser^2^ *α*-CH), 4.53 (1H, *m*, Phe^6^ *α*-CH), 5.04 (1H, *br. s*, Ser^2^ OH), 7.15 (1H, *m*, Phe^6^ CH_Ar_), 7.22 (4H, *m*, Phe^6^ CH_Ar_), 7.75 (1H, *d, J=* 9.0 Hz, Val^4^ NH), 7.93 (1H, *d, J=* 7.2 Hz, Ala^5^ NH), 8.09 (2H, *m*, Leu^1^ NH_2_), 8.20 (1H, *d, J=* 7.2 Hz, Ala^3^ NH), 8.28 (1H, *d, J=* 9.0 Hz, Phe^6^ NH), 8.38 (1H, *t, J=* 6.0 Hz, Gly^7^ NH), 8.67 (1H, *d, J=* 7.8 Hz, Ser^2^ NH); ^13^C-NMR (*d_6_*-DMSO, 150 MHz): *δ* ppm 17.8, 17.9, 18.3, 19.0, 21.8, 22.7, 23.5, 30.7, 37.7, 40.2, 40.7, 48.4, 48.4, 50.7, 53.6, 54.8, 57.4, 61.7, 126.2, 127.9, 129.2, 137.8, 169.0, 169.2, 170.3, 171.0, 171.7, 171.8, 171.9; HR-FAB-MS (M + H)^+^: Calculated for [C_31_H_49_N_7_O_9_ + H]^+^: 664.3670; found: 664.3686.

1.10 L-leucyl-L-seryl-L-alanyl-L-valyl-L-allothreonyl-D-alanylglycine (F6**A**)

Peptide F6**A** (**10**) was synthesized on Wang resin (0.5 g, 1.2 mmol/g). Overall yield: (425 mg, 85 %); t_R_: 2.69 min (ACN:H_2_O:TFA; 60:40:0.08); ^1^H NMR (*d_6_*-DMSO, 600 MHz): 0.82 (3H, *d, J=* 6.6 Hz, Val^4^ γ^1^-CH_3_), 0.84 (3H, *d, J=* 6.6 Hz, Val^4^ γ^2^-CH_3_), 0.87 (3H, *d, J=* 6.6 Hz, Leu^1^ δ^1^-CH_3_), 0.89 (3H, *d, J=* 6.0 Hz, Leu^1^ δ^2^-CH_3_), 1.00 (3H, *d, J=* 6.0 Hz, Thr^5^ γ -CH_3_), 1.20 (6H, *d, J=* 6.6 Hz, Ala^3,6^ *β*-CH_3_), 1.48 (1H, *m*, Leu^1^ *β*-CH_2_), 1.55 (1H, *m*, Leu^1^ *β*-CH_2_), 1.63 (1H, *m*, Leu^1^ *γ*-CH), 1.99 (1H, *m*, Val^4^ *β*-CH), 3.58 (2H, *m*, Ser^2^ *β*-CH_2_), 3.73 (2H, *d, J=* 5.4 Hz, Gly^7^ *α*-CH_2_), 3.84 (1H, *m*, Leu^1^ *α*-CH), 3.94 (1H, *m*, Thr^5^ *β*-CH), 4.13 (1H, *m*, Thr^5^ *α*-CH), 4.21 (1H, *m*, Val^4^ *α*-CH) 4.29 (1H, *m*, Ala^6^ *α*-CH), 4.34 (1H, *m*, Ala^3^ *α*-CH), 4.41 (1H, *m*, Ser^2^ *α*-CH), 5.03 (1H, *br. s*, Thr^5^ OH), 5.09 (1H, *br. s*, Ser^2^ OH), 7.85-7.91 (3H, *m*, Val^4^ NH, Thr^5^ NH, Ala^6^ NH), 8.15 (1H, *d, J=* 7.2 Hz, Ala^3^ NH), 8.20 (1H, *t, J=* 5.4 Hz, Gly^7^ NH), 8.68 (1H, *d, J=* 7.8 Hz, Ser^2^ NH); ^13^C-NMR (*d_6_*-DMSO, 150 MHz): *δ* ppm 18.0, 18.2, 19.1, 19.5, 21.8, 22.6, 23.4, 30.2, 40.1, 40.5, 48.0, 48.3, 50.7, 54.8, 57.7, 58.5, 61.7, 66.3, 169.0, 169.1, 169.6, 170.9, 171.1, 172.1, 172.3; HR-FAB-MS (M + H)^+^: Calculated for [C_26_H_47_N_7_O_10_ + H]^+^: 618.3463; found: 618.3479.

1.11 L-leucyl-L-seryl-L-alanyl-L-valyl-L-allothreonyl-L-phenylalanyl-D-alanine (G7**A**)

Peptide G7**A** (**11**) was synthesized on Wang resin (0.5 g, 1.2 mmol/g). Overall yield: (340 mg, 68 %); t_R_: 2.87 min (ACN:H_2_O:TFA; 60:40:0.08); ^1^H NMR (*d_6_*-DMSO, 600 MHz): 0.77 (3H, *d, J=* 6.6 Hz, Val^4^ γ^1^-CH_3_), 0.79 (3H, *d, J=* 7.2 Hz, Val^4^ γ^2^-CH_3_), 0.87 (3H, *d, J=* 6.6 Hz, Leu^1^ δ^1^-CH_3_), 0.89 (3H, *d, J=* 6.6 Hz, Leu^1^ δ^2^-CH_3_), 0.96 (3H, *d, J=* 6.6 Hz, Thr^5^ γ -CH_3_), 1.16 (3H, *d, J=* 7.2 Hz, Ala^7^ β -CH_3_), 1.20 (3H, *d, J=* 6.6 Hz, Ala^3^ *β*-CH_3_), 1.47 (1H, *m*, Leu^1^ *β*-CH_2_), 1.55 (1H, *m*, Leu^1^ *β*-CH_2_), 1.63 (1H, *m*, Leu^1^ *γ*-CH), 1.94 (1H, *m*, Val^4^ *β*-CH), 2.82 (1H, *dd*, *J=* 8.4, 13.8 Hz, Phe^6^ *β*-CH_2_), 2.98 (1H, *dd*, *J=* 5.4, 13.8 Hz, Phe^6^ *β*-CH_2_), 3.58 (2H, *m*, Ser^2^ *β*-CH_2_), 3.81 (1H, *t*, *J=* 7.2 Hz, Leu^1^ *α*-CH), 3.94 (1H, *m*, Thr^5^ *β*-CH), 4.13 (1H, *m*, Ala^7^ *α*-CH), 4.19-4.24 (2H, *m*, Val^4^ *α*-CH, Thr^5^ *α*-CH), 4.31 (1H, *m*, Ala^3^ *α*-CH), 4.40 (1H, *m*, Ser^2^ *α*-CH), 4.53 (1H, *m*, Phe^6^ *α*-CH), 5.06 (1H, *br. s*, Thr^5^ OH), 5.10 (1H, *br. s*, Ser^2^ OH), 7.15-7.22 (5H, *m*, Phe^6^ CH_Ar_), 7.71 (1H, *d, J=* 8.4 Hz, Thr^5^ NH), 7.87 (1H, *d, J=* 9.0 Hz, Val^4^ NH), 7.91 (1H, *d, J=* 8.4 Hz, Phe^6^ NH), 8.15 (2H, *d, J=* 6.6 Hz, Ala^3,7^ NH), 8.67 (1H, *d, J=* 7.8 Hz, Ser^2^ NH); ^13^C-NMR (*d_6_*-DMSO, 150 MHz): *δ* ppm 17.3, 17.8, 18.0, 19.1, 19.2, 22.7, 23.5, 30.3, 37.6, 40.3, 47.5, 48.3, 50.8, 53.6, 54.8, 57.6, 57.6, 61.7, 66.5, 126.2, 128.0, 129.1, 137.4, 169.1, 169.2, 169.2, 170.1, 170.8, 172.1, 173.9; HR-FAB-MS (M + H)^+^: Calculated for [C_33_H_53_N_7_O_10_ + H]^+^: 708.3932; found: 708.3916.

1.12 L-alanyl-L-seryl-L-alanyl-L-valyl-L-allothreonyl-L-phenylalanylglycine (L1A)

Peptide L1A (**12**) was synthesized on Wang resin (0.5 g, 1.2 mmol/g). Overall yield: (470 mg, 94 %); t_R_: 2.85 min (ACN:H_2_O:TFA; 60:40:0.08); ^1^H NMR (*d_6_*-DMSO, 600 MHz): 0.76 (3H, *d, J=* 7.1 Hz, Val^4^ γ^1^-CH_3_), 0.78 (3H, *d, J=* 7.1 Hz, Val^4^ γ^2^-CH_3_), 0.95 (3H, *d, J=* 6.3 Hz, Thr^5^ γ -CH_3_), 1.20 (3H, *d, J=* 7.0 Hz, Ala^3^ *β*-CH_3_), 1.32 (3H, *d, J=* 6.9 Hz, Ala^1^ *β*-CH_3_), 1.94 (1H, *m*, Val^4^ *β*-CH), 2.82 (1H, *dd*, *J=* 9.1, 14.0 Hz, Phe^6^ *β*-CH_2_), 3.06 (1H, *dd*, *J=* 4.6, 14.1 Hz, Phe^6^ *β*-CH_2_), 3.58 (2H, *m*, Ser^2^ *β*-CH_2_), 3.72 (2H, *m*, Gly^7^ *α*-CH_2_), 3.88 (1H, *m*, Ala^1^ *α*-CH), 3.92 (1H, *m*, Thr^5^ *β*-CH), 4.18-4.20 (2H, *m*, Val^4^ *α*-CH, Thr^5^ *α*-CH), 4.32 (1H, *m*, Ala^3^ *α*-CH), 4.38 (1H, *m*, Ser^2^ *α*-CH), 4.55 (1H, *m*, Phe^6^ *α*-CH), 5.04 (1H, *br. s*, Thr^5^ OH), 5.13 (1H, *br. s*, Ser^2^ OH), 7.15 (1H, *m*, Phe^6^ CH_Ar_), 7.21 (4H, *m*, Phe^6^ CH_Ar_), 7.69 (1H, *d, J=* 8.2 Hz, Thr^5^ NH), 7.87 (1H, *d, J=* 8.8 Hz, Val^4^ NH), 7.91 (1H, *d, J=* 8.2 Hz, Phe^6^ NH), 8.16 (1H, *d, J=* 7.3 Hz, Ala^3^ NH), 8.28 (1H, *t, J=* 5.4 Hz, Gly^7^ NH), 8.57 (1H, *d, J=* 7.8 Hz, Ser^2^ NH); ^13^C-NMR (*d_6_*-DMSO, 150 MHz): *δ* ppm 17.3, 17.8, 18.1, 19.2, 30.3, 37.4, 40.9, 48.1, 48.3, 53.6, 54.9, 57.6, 57.7, 61.7, 66.6, 126.2, 128.0, 129.1, 137.6, 169.2, 169.4, 169.7, 170.8, 171.0, 171.1, 172.1; HR-FAB-MS (M + H)^+^: Calculated for [C_29_H_45_N_7_O_10_ + H]^+^: 652.3306; found: 652.3348.

1.13 Dimethylammonium L-alanyl-L-seryl-L-alanyl-L-valyl-L-allothreonyl-L-phenylalanylglycinate (L1A.DMA)

Peptide L1A.DMA (**13**) was synthesized on Wang resin (0.5 g, 1.2 mmol/g). Overall yield: (450 mg, 90 %); t_R_: 2.75 min (ACN:H_2_O:TFA; 60:40:0.08); ^1^H NMR (*d_6_*-DMSO, 600 MHz): 0.76 (3H, *d, J=* 7.2 Hz, Val^4^ γ^1^-CH_3_), 0.78 (3H, *d, J=* 7.2 Hz, Val^4^ γ^2^-CH_3_), 0.95 (3H, *d, J=* 6.0 Hz, Thr^5^ γ -CH_3_), 1.20 (3H, *d, J=* 7.2 Hz, Ala^3^ *β*-CH_3_), 1.33 (3H, *d, J=* 7.2 Hz, Ala^1^ *β*-CH_3_), 1.94 (1H, *m*, Val^4^ *β*-CH), 2.53 (6H, *s*, dMA CH_3_), 2.81 (1H, *dd*, *J=* 9.0, 14.4 Hz, Phe^6^ *β*-CH_2_), 3.05 (1H, *dd*, *J=* 4.2, 13.8 Hz, Phe^6^ *β*-CH_2_), 3.58 (2H, *m*, Ser^2^ *β*-CH_2_), 3.72 (2H, *t, J=* 5.4 Hz, Gly^7^ *α*-CH_2_), 3.88-3.92 (2H, *m*, Ala^1^ *α*-CH, Thr^5^ *β*-CH) 4.18-4.20 (2H, *m*, Val^4^ *α*-CH, Thr^5^ *α*-CH), 4.32 (1H, *m*, Ala^3^ *α*-CH), 4.38 (1H, *m*, Ser^2^ *α*-CH), 4.55 (1H, *m*, Phe^6^ *α*-CH), 5.02 (1H, *br. s*, Thr^5^ OH), 5.04 (1H, *br. s*, Ser^2^ OH), 7.15 (1H, *m*, Phe^6^ CH_Ar_), 7.21 (4H, *m*, Phe^6^ CH_Ar_), 7.69 (1H, *d, J=* 7.8 Hz, Thr^5^ NH), 7.86 (1H, *d, J=* 9.0 Hz, Val^4^ NH), 7.90 (1H, *d, J=* 8.4 Hz, Phe^6^ NH), 8.14 (1H, *d, J=* 7.2 Hz, Ala^3^ NH), 8.29 (1H, *br. s*, Gly^7^ NH), 8.58 (1H, *d, J=* 7.8 Hz, Ser^2^ NH); ^13^C-NMR (*d_6_*-DMSO, 150 MHz): *δ* ppm 17.3, 17.9, 18.1, 19.2, 30.4, 34.3, 37.4, 40.8, 48.0, 48.3, 53.6, 55.0, 57.6, 57.7, 61.7, 66.6, 126.2, 128.0, 129.1, 137.5, 169.2, 169.4, 169.5, 170.8, 171.0, 171.1, 172.1; HR-FAB-MS (M + H)^+^: Calculated for [C_29_H_45_N_7_O_10_ + H]^+^: 652.3306; found: 652.3292.

1.14 L-leucyl-L-alanyl-L-alanyl-L-valyl-L-allothreonyl-L-phenylalanylglycine (S2A)

Peptide S2A (**14**) was synthesized on Wang resin (0.5 g, 1.2 mmol/g). Overall yield: (450 mg, 90 %); t_R_: 2.94 min (ACN:H_2_O:TFA; 60:40:0.08); ^1^H NMR (*d_6_*-DMSO, 600 MHz): 0.76 (3H, *d, J=* 7.2 Hz, Val^4^ γ^1^-CH_3_), 0.78 (3H, *d, J=* 6.6 Hz, Val^4^ γ^2^-CH_3_), 0.86 (3H, *d, J=* 6.0 Hz, Leu^1^ δ^1^-CH_3_), 0.89 (3H, *d, J=* 6.6 Hz, Leu^1^ δ^2^-CH_3_), 0.95 (3H, *d, J=* 6.6 Hz, Thr^5^ *γ*-CH_3_), 1.18 (3H, *d, J=* 6.6 Hz, Ala^3^ *β*-CH_3_), 1.23 (3H, *d, J=* 6.6 Hz, Ala^2^ *β*-CH_3_), 1.47 (1H, *m*, Leu^1^ *β*-CH_2_), 1.52 (1H, *m*, Leu^1^ *β*-CH_2_), 1.63 (1H, *m*, Leu^1^ *γ*-CH), 1.95 (1H, *m*, Val^4^ *β*-CH), 2.81 (1H, *dd*, *J=* 9.0, 13.8 Hz, Phe^6^ *β*-CH_2_), 3.05 (1H, *dd*, *J=* 4.2, 13.8 Hz, Phe^6^ *β*-CH_2_), 3.73 (2H, *m*, Gly^7^ *α*-CH_2_), 3.76 (1H, *m*, Leu^1^ *α*-CH), 3.92 (1H, *m*, Thr^5^ *β*-CH), 4.18-4.21 (2H, *m*, Val^4^ *α*-CH, Thr^5^ *α*-CH), 4.31 (1H, *m*, Ala^3^ *α*-CH), 4.38 (1H, *m*, Ala^2^ *α*-CH), 4.55 (1H, *m*, Phe^6^ *α*-CH), 4.98 (1H, *br. s*, Thr^5^ OH), 7.15 (1H, *m*, Phe^6^ CH_Ar_), 7.21 (4H, *m*, Phe^6^ CH_Ar_), 7.68 (1H, *d, J=* 8.4 Hz, Thr^5^ NH), 7.75 (1H, *d, J=* 9.0 Hz, Val^4^ NH), 7.89 (1H, *d, J=* 8.4 Hz, Phe^6^ NH), 8.12 (1H, *d, J=* 7.2 Hz, Ala^3^ NH), 8.32 (1H, *t, J=* 5.4 Hz, Gly^7^ NH), 8.65 (1H, *d, J=* 7.2 Hz, Ala^2^ NH); ^13^C-NMR (*d_6_*-DMSO, 150 MHz): *δ* ppm 17.7, 17.9, 18.2, 19.1, 19.1, 21.8, 22.6, 23.4, 30.5, 37.4, 40.2, 40.7, 48.0, 48.1, 50.7, 53.6, 57.4, 57.6, 66.6, 126.2, 128.0, 129.1, 137.5, 168.5, 169.4, 170.6, 171.0, 171.1, 171.2, 171.9; HR-FAB-MS (M + H)^+^: Calculated for [C_32_H_51_N_7_O_9_ + H]^+^: 678.3827; found: 678.3781.

1.15 Dimethylammonium L-leucyl-L-alanyl-L-alanyl-L-valyl-L-allothreonyl-L-phenylalanylglycinate (S2A.DMA)

Peptide S2A.DMA (**15**) was synthesized on Wang resin (0.5 g, 1.2 mmol/g). Overall yield: (440 mg, 88 %); t_R_: 2.76 min (ACN:H_2_O:TFA; 60:40:0.08); ^1^H NMR (*d_6_*-DMSO, 600 MHz): 0.76 (3H, *d, J=* 7.2 Hz, Val^4^ γ^1^-CH_3_), 0.78 (3H, *d, J=* 6.6 Hz, Val^4^ γ^2^-CH_3_), 0.87 (3H, *d, J=* 6.0 Hz, Leu^1^ δ^1^-CH_3_), 0.89 (3H, *d, J=* 6.6 Hz, Leu^1^ δ^2^-CH_3_), 0.95 (3H, *d, J=* 6.6 Hz, Thr^5^ *γ*-CH_3_), 1.18 (3H, *d, J=* 6.6 Hz, Ala^3^ *β*-CH_3_), 1.23 (3H, *d, J=* 6.6 Hz, Ala^2^ *β*-CH_3_), 1.48 (1H, *m*, Leu^1^ *β*-CH_2_), 1.52 (1H, *m*, Leu^1^ *β*-CH_2_), 1.63 (1H, *m*, Leu^1^ *γ*-CH), 1.95 (1H, *m*, Val^4^ *β*-CH), 2.53 (6H, *s*, dMA CH_3_), 2.81 (1H, *dd*, *J=* 9.0, 13.8 Hz, Phe^6^ *β*-CH_2_), 3.05 (1H, *dd*, *J=* 4.2, 13.8 Hz, Phe^6^ *β*-CH_2_), 3.73 (2H, *t, J=* 5.4 Hz, Gly^7^ *α*-CH_2_), 3.76 (1H, *m*, Leu^1^ *α*-CH), 3.92 (1H, *m*, Thr^5^ *β*-CH), 4.18-4.21 (2H, *m*, Val^4^ *α*-CH, Thr^5^ *α*-CH), 4.31 (1H, *m*, Ala^3^ *α*-CH), 4.38 (1H, *m*, Ala^2^ *α*-CH), 4.55 (1H, *m*, Phe^6^ *α*-CH), 4.98 (1H, *br. s*, Thr^5^ OH), 7.15 (1H, *m*, Phe^6^ CH_Ar_), 7.21 (4H, *m*, Phe^6^ CH_Ar_), 7.68 (1H, *d, J=* 8.4 Hz, Thr^5^ NH), 7.75 (1H, *d, J=* 9.0 Hz, Val^4^ NH), 7.90 (1H, *d, J=* 8.4 Hz, Phe^6^ NH), 8.13 (1H, *d, J=* 7.2 Hz, Ala^3^ NH), 8.31 (1H, *t, J=* 5.4 Hz, Gly^7^ NH), 8.65 (1H, *d, J=* 7.2 Hz, Ala^2^ NH); ^13^C-NMR (*d_6_*-DMSO, 150 MHz): *δ* ppm 17.7, 17.9, 18.2, 19.1, 19.1, 21.8, 22.6, 23.4, 30.5, 34.2, 37.4, 40.2, 40.7, 48.0, 48.1, 50.7, 53.5, 57.4, 57.6, 66.5, 126.2, 128.0, 129.1, 137.5, 168.5, 169.4, 170.7, 170.9, 171.0, 171.2, 171.9; HR-FAB-MS (M + H)^+^: Calculated for [C_32_H_51_N_7_O_9_ + H]^+^: 678.3827; found: 678.3799.

1.16 Dimethylammonium L-leucyl-L-seryl-L-alanyl-L-alanyl-L-allothreonyl-L-phenylalanylglycinate (V4A.DMA)

Peptide V4A.DMA (**16**) was synthesized on Wang resin (0.5 g, 1.2 mmol/g). Overall yield: (335 mg, 67 %); t_R_: 2.60 min (ACN:H_2_O:TFA; 60:40:0.08); ^1^H NMR (*d_6_*-DMSO, 600 MHz): 0.86 (3H, *d, J=* 6.0 Hz, Leu^1^ δ^1^-CH_3_), 0.88 (3H, *d, J=* 6.0 Hz, Leu^1^ δ^2^-CH_3_), 0.94 (3H, *d, J=* 6.0 Hz, Thr^5^ *γ*-CH_3_), 1.17 (3H, *d, J=* 6.0 Hz, Ala^4^ *β*-CH_3_), 1.21 (3H, *d, J=* 6.0 Hz, Ala^3^ *β*-CH_3_), 1.50 (1H, *m*, Leu^1^ *β*-CH_2_), 1.54 (1H, *m*, Leu^1^ *β*-CH_2_), 1.63 (1H, *m*, Leu^1^ *γ*-CH), 2.53 (6H, *s*, dMA CH_3_), 2.85 (1H, *dd*, *J=* 9.0, 13.8 Hz, Phe^6^ *β*-CH_2_), 3.09 (1H, *dd*, *J=* 4.2, 14.4 Hz, Phe^6^ *β*-CH_2_), 3.55 (1H, *dd*, *J=* 6.0, 12.0 Hz, Ser^2^ *β*-CH_2_), 3.65 (1H, *dd*, *J=* 6.0, 12.0 Hz, Ser^2^ *β*-CH_2_), 3.71 (2H, *d, J=* 6.0 Hz, Gly^7^ *α*-CH_2_), 3.82 (1H, *m*, Leu^1^ *α*-CH), 3.95 (1H, *m*, Thr^5^ *β*-CH), 4.15 (1H, *m*, Thr^5^ *α*-CH), 4.25 (1H, *m,*, Ala^3^ *α*-CH), 4.31 (1H, *m,* Ala^4^ *α*-CH), 4.41 (1H, *m,* Ser^2^ *α*-CH), 4.51 (1H, *m*, Phe^6^ *α*-CH), 7.17 (1H, *m*, Phe^6^ CH_Ar_), 7.23 (4H, *m*, Phe^6^ CH_Ar_), 7.80 (1H, *d, J=* 6.0 Hz, Thr^5^ NH), 7.97 (2H, *d, J=* 6.0 Hz, Phe^6^ NH, Ala^4^ NH), 8.15 (1H, *br. s*, Gly^7^ NH), 8.34 (1H, *d, J=* 6.0 Hz, Ala^3^ NH), 8.71 (1H, *d, J=* 12.0 Hz, Ser^2^ NH); ^13^C-NMR (*d_6_*-DMSO, 150 MHz): *δ* ppm 17.8, 18.1, 19.2, 21.8, 22.6, 23.5, 34.2, 37.1, 40.2, 40.9, 48.5, 48.5, 50.8, 54.0, 54.7, 57.9, 61.7, 66.3, 126.2, 128.0, 129.1, 137.8, 169.2, 169.3, 169.5, 171.0, 171.1, 171.9, 172.4; HR-FAB-MS (M + H)^+^: Calculated for [C_30_H_47_N_7_O_10_ + H]^+^: 666.3463; found: 666.3433.

1.17 Dimethylammonium L-leucyl-L-seryl-L-alanyl-L-valyl-L-alanyl-L-phenylalanylglycinate (T5A.DMA)

Peptide T5A.DMA (**17**) was synthesized on Wang resin (0.5 g, 1.2 mmol/g). Overall yield: (200 mg, 40 %); t_R_: 2.72 min (ACN:H_2_O:TFA; 60:40:0.08); ^1^H NMR (*d_6_*-DMSO, 600 MHz): 0.75 (3H, *d, J=* 6.6 Hz, Val^4^ γ^1^-CH_3_), 0.78 (3H, *d, J=* 6.6 Hz, Val^4^ γ^2^-CH_3_), 0.86 (3H, *d, J=* 6.6 Hz, Leu^1^ δ^1^-CH_3_), 0.88 (3H, *d, J=* 6.0 Hz, Leu^1^ δ^2^-CH_3_), 1.14 (3H, *d, J=* 7.2 Hz, Ala^5^ *β*-CH_3_), 1.20 (3H, *d, J=* 7.2 Hz, Ala^3^ *β*-CH_3_), 1.47 (1H, *m*, Leu^1^ *β*-CH_2_), 1.55 (1H, *m*, Leu^1^ *β*-CH_2_), 1.63 (1H, *m*, Leu^1^ *γ*-CH), 1.90 (1H, *m*, Val^4^ *β*-CH), 2.53 (6H, *s*, dMA CH_3_), 2.80 (1H, *dd*, *J=* 9.6, 14.4 Hz, Phe^6^ *β*-CH_2_), 3.01 (1H, *dd*, *J=* 4.2, 13.8 Hz, Phe^6^ *β*-CH_2_), 3.58 (2H, *m*, Ser^2^ *β*-CH_2_), 3.72 (2H, *m*, Gly^7^ *α*-CH_2_), 3.82 (1H, *m*, Leu^1^ *α*-CH), 4.12 (1H, *m*, Val^4^ *α*-CH), 4.21 (1H, *m*, Ala^5^ *α*-CH), 4.30 (1H, *m*, Ala^3^ *α*-CH), 4.40 (1H, *m*, Ser^2^ *α*-CH), 4.49 (1H, *m*, Phe^6^ *α*-CH), 7.15 (1H, *m*, Phe^6^ CH_Ar_), 7.21 (4H, *m*, Phe^6^ CH_Ar_), 7.75 (1H, *d, J=* 9.0 Hz, Val^4^ NH), 7.93 (1H, *d, J=* 6.6 Hz, Ala^5^ NH), 7.94 (1H, *d, J=* 7.8 Hz, Phe^6^ NH), 8. 20 (1H, *m,* Gly^7^ NH), 8.21 (1H, *d, J=* 7.2 Hz, Ala^3^ NH), 8.69 (1H, *d, J=* 7.8 Hz, Ser^2^ NH); ^13^C-NMR (*d_6_*-DMSO, 150 MHz): *δ* ppm 17.8, 17.9, 18.1, 19.2, 21.8, 22.7, 23.5, 30.6, 34.3, 37.5, 40.3, 40.9, 48.2, 48.5, 50.8, 53.7, 54.9, 57.4, 61.8, 126.2, 128.0, 129.2, 137.6, 169.2, 169.3, 170.4, 171.1, 171.2, 171.8, 172.1; HR-FAB-MS (M + H)^+^: Calculated for [C_31_H_49_N_7_O_9_ + H]^+^: 664.3670; found: 664.3647.

1.18 Dimethylammonium L-leucyl-L-seryl-L-alanyl-L-valyl-L-allothreonyl-L-alanylglycinate (F6A.DMA)

Peptide F6A.DMA (**18**) was synthesized on Wang resin (0.5 g, 1.2 mmol/g). Overall yield: (315 mg, 63 %); t_R_: 2.49 min (ACN:H_2_O:TFA; 60:40:0.08); ^1^H NMR (*d_6_*-DMSO, 600 MHz): 0.81 (3H, *d, J=* 6.6 Hz, Val^4^ γ^1^-CH_3_), 0.84 (3H, *d, J=* 6.6 Hz, Val^4^ γ^2^-CH_3_), 0.87 (3H, *d, J=* 6.3 Hz, Leu^1^ δ^1^-CH_3_), 0.89 (3H, *d, J=* 6.4 Hz, Leu^1^ δ^2^-CH_3_), 1.00 (3H, *d, J=* 6.0 Hz, Thr^5^ γ -CH_3_), 1.21 (6H, *d, J=* 6.6 Hz, Ala^3,6^ *β*-CH_3_), 1.48 (1H, *m*, Leu^1^ *β*-CH_2_), 1.55 (1H, *m*, Leu^1^ *β*-CH_2_), 1.63 (1H, *m*, Leu^1^ *γ*-CH), 1.99 (1H, *m*, Val^4^ *β*-CH), 2.53 (2H, *s*, dMA CH_3_), 3.58 (2H, *m*, Ser^2^ *β*-CH_2_), 3.70 (2H, *d, J=* 5.4 Hz, Gly^7^ *α*-CH_2_), 3.82 (1H, *m*, Leu^1^ *α*-CH), 3.99 (1H, *m*, Thr^5^ *β*-CH), 4.19-4.23 (2H, *m*, Thr^5^ *α*-CH), Val^4^ *α*-CH) 4.29 (1H, *m*, Ala^6^ *α*-CH), 4.33 (1H, *m*, Ala^3^ *α*-CH), 4.40 (1H, *m*, Ser^2^ *α*-CH), 5.09 (1H, *br. s*, Ser^2^ OH), 7.74 (1H, *d, J=* 6.8 Hz, Val^4^ NH), 7.90 (1H, *d, J=* 7.3 Hz, Ala^6^ NH), 1.00 (1H, *d, J=* 5.2 Hz, Thr^5^ NH), 8.11 (1H, *t, J=* 5.4 Hz, Gly^7^ NH), 8.16 (1H, *d, J=* 6.0 Hz, Ala^3^ NH), 8.69 (1H, *d, J=* 6.4 Hz, Ser^2^ NH); ^13^C-NMR (*d_6_*-DMSO, 150 MHz): *δ* ppm 17.9, 18.0, 18.2, 19.2, 19.3, 21.8, 22.7, 23.5, 30.3, 34.2, 40.3, 40.7, 48.0, 48.3, 50.8, 54.9, 57.7, 57.8, 61.7, 66.5, 169.1, 169.2, 169.2, 170.9, 171.1, 172.1, 172.2; HR-FAB-MS (M + H)^+^: Calculated for [C_26_H_47_N_7_O_10_ + H]^+^: 618.3463; found: 618.3445.

1.19 Dimethylammonium L-leucyl-L-seryl-L-alanyl-L-valyl-L-allothreonyl-L-phenylalanyl-L-alaninate (G7A.DMA)

Peptide G7A.DMA (**19**) was synthesized on Wang resin (0.5 g, 1.2 mmol/g). Overall yield: (445 mg, 89 %); t_R_: 2.71 min (ACN:H_2_O:TFA; 60:40:0.08); ^1^H NMR (*d_6_*-DMSO, 600 MHz): 0.76 (3H, *d, J=* 6.6 Hz, Val^4^ γ^1^-CH_3_), 0.78 (3H, *d, J=* 7.2 Hz, Val^4^ γ^2^-CH_3_), 0.87 (3H, *d, J=* 6.6 Hz, Leu^1^ δ^1^-CH_3_), 0.89 (3H, *d, J=* 6.6 Hz, Leu^1^ δ^2^-CH_3_), 0.95 (3H, *d, J=* 6.6 Hz, Thr^5^ γ -CH_3_), 1.19 (3H, *d, J=* 7.2 Hz, Ala^7^ β -CH_3_), 1.26 (3H, *d, J=* 6.6 Hz, Ala^3^ *β*-CH_3_), 1.48 (1H, *m*, Leu^1^ *β*-CH_2_), 1.55 (1H, *m*, Leu^1^ *β*-CH_2_), 1.63 (1H, *m*, Leu^1^ *γ*-CH), 1.94 (1H, *m*, Val^4^ *β*-CH), 2.53 (2H, *s*, dMA CH_3_), 2.78 (1H, *dd*, *J=* 8.4, 13.8 Hz, Phe^6^ *β*-CH_2_), 3.06 (1H, *dd*, *J=* 5.4, 13.8 Hz, Phe^6^ *β*-CH_2_), 3.58 (2H, *m*, Ser^2^ *β*-CH_2_), 3.83 (1H, *m*, Leu^1^ *α*-CH), 3.89 (1H, *m*, Thr^5^ *β*-CH), 4.16-4.20 (3H, *m*, Ala^7^ *α*-CH, Val^4^ *α*-CH, Thr^5^ *α*-CH), 4.32 (1H, *m*, Ala^3^ *α*-CH), 4.40 (1H, *m*, Ser^2^ *α*-CH), 4.53 (1H, *m*, Phe^6^ *α*-CH), 4.97 (1H, *br. s*, Thr^5^ OH), 5.09 (1H, *br. s*, Ser^2^ OH), 7.14 (1H, *m*, Phe^6^ CH_Ar_), 7.21 (4H, *m*, Phe^6^ CH_Ar_), 7.69 (1H, *d, J=* 8.2 Hz, Thr^5^ NH), 7.83 (1H, *d, J=* 8.3 Hz, Phe^6^ NH), 7.85 (1H, *d, J=* 8.5 Hz, Val^4^ NH), 8.15 (1H, *d, J=* 6.5 Hz, Ala^3^ NH), 8.28 (1H, *d, J=* 6.8 Hz, Ala^7^ NH), 8.68 (1H, *d, J=* 7.5 Hz, Ser^2^ NH); ^13^C-NMR (*d_6_*-DMSO, 150 MHz): *δ* ppm 17.0, 17.8, 18.0, 19.1, 19.2, 21.8, 22.7, 23.5, 30.4, 34.3, 37.2, 40.2, 47.5, 48.3, 50.7, 53.3, 54.9, 57.6, 57.7, 61.7, 66.5, 126.2, 128.0, 129.1, 137.5, 169.0, 169.1, 169.3, 170.5, 170.7, 172.1, 173.8; HR-FAB-MS (M + H)^+^: Calculated for [C_33_H_53_N_7_O_10_ + H]^+^: 708.3932; found: 708.3976.

Figure-S1: HR-FAB-MS (positive mode) of peptide LP (**1**)

Figure-S2: ^1^H-NMR (600 MHz) spectra of peptide LP (**1**) in DMSO-*d6*.

Figure-S3: HSQC correlations of peptide LP (**1**)

Figure-S4: COSY correlations of peptide LP (**1**)

Figure-S5: BB of peptide LP (**1**)

Figure-S6: HR-FAB-MS (positive mode) of peptide P6F (**2**)

Figure-S7: ^1^H-NMR (600 MHz) spectra of peptide P6F (**2**) in DMSO-*d6*.

Figure-S8: HSQC correlations of peptide P6F (**2**)

Figure-S9: COSY correlations of peptide P6F (**2**)

Figure-S10: BB of peptide P6F (**2**)

Figure-S11: HR-FAB-MS (positive mode) of peptide P6F.DMA (**3**)

Figure-S12: ^1^H-NMR (600 MHz) spectra of peptide P6F.DMA (**3**) in DMSO-*d6*.

Figure-S13: HSQC correlations of peptide P6F.DMA (**3**)

Figure-S14: COSY correlations of peptide P6F.DMA (**3**)

Figure-S15: HMBC correlations of peptide P6F.DMA (**3**)

Figure-S16: DEPT 135 of peptide P6F.DMA (**3**)

Figure-S17: DEPT 90 of peptide P6F.DMA (**3**)

Figure-S18: BB of peptide P6F.DMA (**3**)

Figure-S19: DOSY spectra of peptide P6F.DMA (**3**)

Figure-S20: HR-FAB-MS (positive mode) of peptide L1**A** (**4**)

Figure-S21: ^1^H-NMR (600 MHz) spectra of peptide L1**A** (**4**) in DMSO-*d6*.

Figure-S22: HSQC correlations of peptide L1**A** (**4**)

Figure-S23: COSY correlations of peptide L1**A** (**4**)

Figure-S24: BB of peptide L1**A** (**4**)

Figure-S25: HR-FAB-MS (positive mode) of peptide L1**A**.DMA (**5**)

Figure-S26: ^1^H-NMR (600 MHz) spectra of peptide L1**A**.DMA (**5**) in DMSO-*d6*.

Figure-S27: HSQC correlations of peptide L1**A**.DMA (**5**)

Figure-S28: COSY correlations of peptide L1**A**.DMA (**5**)

Figure-S29: BB of peptide L1**A**.DMA (**5**)

Figure-S30: DOSY spectra of peptide L1**A**.DMA (**5**)

Figure-S31: HR-FAB-MS (positive mode) of peptide S2**A** (**6**)

Figure-S32: ^1^H-NMR (600 MHz) spectra of peptide S2**A** (**6**) in DMSO-*d6*.

Figure-S33: HSQC correlations of peptide S2**A** (**6**)

Figure-S34: COSY correlations of peptide S2**A** (**6**)

Figure-S35: BB of peptide S2**A** (**6**)

Figure-S36: HR-FAB-MS (positive mode) of peptide S2**A**.DMA (**7**)

Figure-S37: ^1^H-NMR (600 MHz) spectra of peptide S2**A**.DMA (**7**) in DMSO-*d6*.

Figure-S38: HSQC correlations of peptide S2**A**.DMA (**7**)

Figure-S39: COSY correlations of peptide S2**A**.DMA (**7**)

Figure-S40: BB of peptide S2**A**.DMA (**7**)

Figure-S41: DOSY spectra of peptide S2**A**.DMA (**7**)

Figure-S42: HR-FAB-MS (positive mode) of peptide V4**A** (**8**)

Figure-S43: ^1^H-NMR (600 MHz) spectra of peptide V4**A** (**8**) in DMSO-*d6*.

Figure-S44: HSQC correlations of peptide V4**A** (**8**)

Figure-S45: COSY correlations of peptide V4**A** (**8**)

Figure-46: BB of peptide V4**A** (**8**)

Figure-S47: HR-FAB-MS (positive mode) of peptide T5**A** (**9**)

Figure-S48: ^1^H-NMR (600 MHz) spectra of peptide T5**A** (**9**) in DMSO-*d6*.

Figure-S49: HSQC correlations of peptide T5**A** (**9**)

Figure-S50: COSY correlations of peptide T5**A** (**9**)

Figure-S51: BB of peptide T5**A** (**9**)

Figure-S52: HR-FAB-MS (positive mode) of peptide F6**A** (**10**)

Figure-S53: ^1^H-NMR (600 MHz) spectra of peptide F6**A** (**10**) in DMSO-*d6*.

Figure-S54: HSQC correlations of peptide F6**A** (**10**)

Figure-S55: COSY correlations of peptide F6**A** (**10**)

Figure-S56: BB of peptide F6**A** (**10**)

Figure-S57: HR-FAB-MS (positive mode) of peptide G7**A** (**11**)

Figure-S58: ^1^H-NMR (600 MHz) spectra of peptide G7**A** (**11**) in DMSO-*d6*.

Figure-S59: HSQC correlations of peptide G7**A** (**11**)

Figure-S60: COSY correlations of peptide G7**A** (**11**)

Figure-S61: BB of peptide G7**A** (**11**)

Figure-S62: HR-FAB-MS (positive mode) of peptide L1A (**12**)

Figure-S63: ^1^H-NMR (600 MHz) spectra of peptide L1A (**12**) in DMSO-*d6*.

Figure-S64: HSQC correlations of peptide L1A (**12**)

Figure-S65: COSY correlations of peptide L1A (**12**)

Figure-S66: HMBC correlations of peptide L1A (**12**)

Figure-S67: DEPT 135 of peptide L1A (**12**)

Figure-S68: DEPT 90 of peptide L1A (**12**)

Figure-S69: BB of peptide L1A (**12**)

Figure-S70: HR-FAB-MS (positive mode) of peptide L1A.DMA (**13**)

Figure-S71: ^1^H-NMR (600 MHz) spectra of peptide L1A.DMA (**13**) in DMSO-*d6*.

Figure-S72: HSQC correlations of peptide L1A.DMA (**13**)

Figure-S73: COSY correlations of peptide L1A.DMA (**13**)

Figure-S74: HMBC correlations of peptide L1A.DMA (**13**)

Figure-S75: DEPT 135 of peptide L1A.DMA (**13**)

Figure-S76: DEPT 90 of peptide L1A.DMA (**13**)

Figure-S77: BB of peptide L1A.DMA (**13**)

Figure-S78: DOSY spectra of peptide L1A.DMA (**13**)

Figure-S79: HR-FAB-MS (positive mode) of peptide S2A (**14**)

Figure-S80: ^1^H-NMR (600 MHz) spectra of peptide S2A (**14**) in DMSO-*d6*.

Figure-S81: HSQC correlations of peptide S2A (**14**)

Figure-S82: COSY correlations of peptide S2A (**14**)

Figure-S83: HMBC correlations of peptide S2A (**14**)

Figure-S84: DEPT 135 of peptide S2A (**14**)

Figure-S85: DEPT 90 of peptide S2A (**14**)

Figure-S86: BB of peptide S2A (**14**)

Figure-S87: HR-FAB-MS (positive mode) of peptide S2A.DMA (**15**)

Figure-S88: ^1^H-NMR (600 MHz) spectra of peptide S2A.DMA (**15**) in DMSO-*d6*.

Figure-S89: HSQC correlations of peptide S2A.DMA (**15**)

Figure-S90: COSY correlations of peptide S2A.DMA (**15**)

Figure-S91: HMBC correlations of peptide S2A.DMA (**15**)

Figure-S92: DEPT 135 of peptide S2A.DMA (**15**)

Figure-S93: DEPT 90 of peptide S2A.DMA (**15**)

Figure-S94: BB of peptide S2A.DMA (**15**)

Figure-S95: DOSY spectra of peptide S2A.DMA (**15**)

Figure-S96: HR-FAB-MS (positive mode) of peptide V4A.DMA (**16**)

Figure-S97: ^1^H-NMR (600 MHz) spectra of peptide V4A.DMA (**16**) in DMSO-*d6*.

Figure-S98: HSQC correlations of peptide V4A.DMA (**16**)

Figure-S99: COSY correlations of peptide V4A.DMA (**16**)

Figure-S100: BB of peptide V4A.DMA (**16**)

Figure-S101: DOSY spectra of peptide V4A.DMA (**16**)

Figure-S102: HR-FAB-MS (positive mode) of peptide T5A.DMA (**17**)

Figure-S103: ^1^H-NMR (600 MHz) spectra of peptide T5A.DMA (**17**) in DMSO-*d6*.

Figure-S104: HSQC correlations of peptide T5A.DMA (**17**)

Figure-S105: COSY correlations of peptide T5A.DMA (**17**)

Figure-S106: BB of peptide T5A.DMA (**17**)

Figure-S107: DOSY spectra of peptide T5A.DMA (**17**)

Figure-S108: HR-FAB-MS (positive mode) of peptide F6A.DMA (**18**)

Figure-S109: ^1^H-NMR (600 MHz) spectra of peptide F6A.DMA (**18**) in DMSO-*d6*.

Figure-S110: HSQC correlations of peptide F6A.DMA (**18**)

Figure-S111: COSY correlations of peptide F6A.DMA (**18**)

Figure-S112: BB of peptide F6A.DMA (**18**)

Figure-S113: DOSY spectra of peptide F6A.DMA (**18**)

Figure-S114: HR-FAB-MS (positive mode) of peptide G7A.DMA (**19**)

Figure-S115: ^1^H-NMR (600 MHz) spectra of peptide G7A.DMA (**19**) in DMSO-*d6*.

Figure-S116: HSQC correlations of peptide G7A.DMA (**19**)

Figure-S117: COSY correlations of peptide G7A.DMA (**19**)

Figure-S118: BB of peptide G7A.DMA (**19**)

Figure-S119: DOSY spectra of peptide G7A.DMA (**19**)

**Figure-S120**

**Figure-S121**
